# Tailoring On-Surface Molecular Reactions and Assembly through Hydrogen-Modified Synthesis: From Triarylamine Monomer to 2D Covalent Organic Framework

**DOI:** 10.1021/acsnano.2c11463

**Published:** 2023-04-04

**Authors:** Zachery A. Enderson, Harshavardhan Murali, Raghunath R. Dasari, Qingqing Dai, Hong Li, Timothy C. Parker, Jean-Luc Brédas, Seth R. Marder, Phillip N. First

**Affiliations:** † School of Physics, Georgia Institute of Technology, Atlanta, Georgia 30332, United States; ‡ School of Chemistry and Biochemistry, Georgia Institute of Technology, Atlanta, Georgia 30332, United States; ¶ Department of Chemistry and Biochemistry, The University of Arizona, Tucson, Arizona 85721, United States; § Department of Chemical and Biological Engineering, Department of Chemistry, and Materials Science and Engineering Program, University of Colorado Boulder, Renewable and Sustainable Energy Institute, Boulder, Colorado 80303, United States; ∥ National Renewable Energy Laboratory, Chemistry and Nanoscience Center, Golden, Colorado 80401, United States

**Keywords:** scanning tunneling microscopy (STM), covalent organic framework (COF), triangulene, heterotriangulene, DTPA, self-assembled monolayer (SAM)

## Abstract

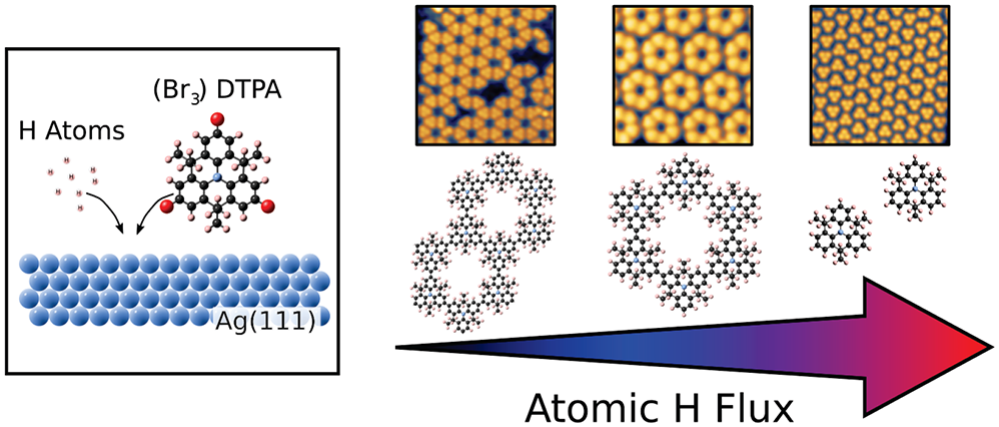

Relative to conventional wet-chemical synthesis techniques, on-surface synthesis of organic networks in ultrahigh vacuum has few control parameters. The molecular deposition rate and substrate temperature are typically the only synthesis variables to be adjusted dynamically. Here we demonstrate that reducing conditions in the vacuum environment can be created and controlled without dedicated sources—relying only on backfilled hydrogen gas and ion gauge filaments—and can dramatically influence the Ullmann-like on-surface reaction used for synthesizing two-dimensional covalent organic frameworks (2D COFs). Using tribromo dimethylmethylene-bridged triphenylamine ((Br_3_)DTPA) as monomer precursors, we find that atomic hydrogen (H^•^) blocks aryl–aryl bond formation to such an extent that we suspect this reaction may be a factor in limiting the ultimate size of 2D COFs created through on-surface synthesis. Conversely, we show that control of the relative monomer and hydrogen fluxes can be used to produce large self-assembled islands of monomers, dimers, or macrocycle hexamers, which are of interest in their own right. On-surface synthesis of oligomers, from a single precursor, circumvents potential challenges with their protracted wet-chemical synthesis and with multiple deposition sources. Using scanning tunneling microscopy and spectroscopy (STM/STS), we show that changes in the electronic states through this oligomer sequence provide an insightful view of the 2D COF (synthesized in the absence of atomic hydrogen) as the end point in an evolution of electronic structures from the monomer.

## Introduction

The targeted synthesis of materials with designed properties or electronic structure is a central goal of materials research. For ordered covalent organic framework (COF) materials, limiting the chemical synthesis to two dimensions (2D) is especially challenging, but its mastery would advance technologies for 2D organic electronics^1−3^ and for membranes used both structurally and for chemical separations.^4^ One approach to fabricating low-dimensional COFs is on-surface synthesis: a version of reticular synthesis^5^ conducted *in vacuo* on a clean surface. Rigid molecular precursors are vapor-deposited onto a substrate, generally metallic, which facilitates bond formation between molecular units.^6^ This method allows for COFs to be carefully designed with specific lattice structures, functional groups, and electronic structure.^7^ In this work, we focus on heterotriangulene 2D polymers from tribromo-substituted dimethylmethylene-bridged triphenylamine ((Br_3_)DTPA) monomer precursors, first studied by Bieri *etal*.^8^ Interest in heterotriangulene COFs arises from their potential optoelectronic and transport properties, which are chemically tunable through their electronic structure: The bandgap and the occurrence of both flat bands (with potential to host correlated states) and linearly dispersive (Dirac) bands are controlled by modifications of the bridge-site moieties or the central heteroatom site.^9−16^ In addition, for a bridge moiety resulting in an open-shell configuration, the 2D COF is predicted to have a fully spin-polarized band structure near the Fermi energy, *E*_F_.^10^

While on-surface synthesis is a valuable approach to create low-dimensional COFs, it is not without limitations. The formation of effectively irreversible covalent bonds makes the extent and the order of 2D COFs highly dependent on the deposition parameters and other environmental factors.^17−20^ In particular, it has been suggested^21^ and shown by scanning probe microscopy^22−25^ that the presence of atomic hydrogen during deposition can inhibit C–C bond formation. Here we use time-of-flight secondary-ion mass spectroscopy (TOF-SIMS) to provide conclusive evidence for the nature of atomic hydrogen’s inhibitory effect on COF growth. We also show that the influence of atomic hydrogen during the formation of 2D COFs can be pervasive: Hot filaments *anywhere* within a vacuum chamber can potentially crack H_2_. Then, from a broader perspective, we demonstrate that atomic hydrogen, even with ambient cracking filaments, could become a valuable reducing agent for control of surface chemistry, enabling the on-surface creation of single- and multimonomer compounds from a single precursor source. Our scanning tunneling microscopy/spectroscopy (STM/STS) investigation of the evolution of electronic structures through such a series of oligomers (monomer, dimer, hexamer macrocycle) provides a deeper understanding of the 2D COF created from the same molecular precursor.

## Results and Discussion

### Hydrogen Effect

Prior work^8,12,26−29^ has established a general understanding of the on-surface reactions for halogen-terminated heterotriangulene monomer precursors. Above 330 K the molecules dehalogenate through an Ullmann-like reaction with the Ag(111) substrate, temporarily leaving them as surface-stabilized radicals.^27^ On Ag(111), surface diffusion enables these species to form organometallic networks with monomer–Ag–monomer linkages. Some examples include the honeycomb organometallic lattice of DTPA (formed after depositing precursors at 473 K)^8^ and 1,3,5-tris(4-bromophenyl)benzene (375 K).^29^ When heated to higher temperatures (500 K or higher as suggested by XPS studies^29^), the molecules have enough energy to complete the Ullmann coupling, forming C–C bonds at their debrominated sites and creating a honeycomb-lattice 2D COF.^8^

**Figure 1:**
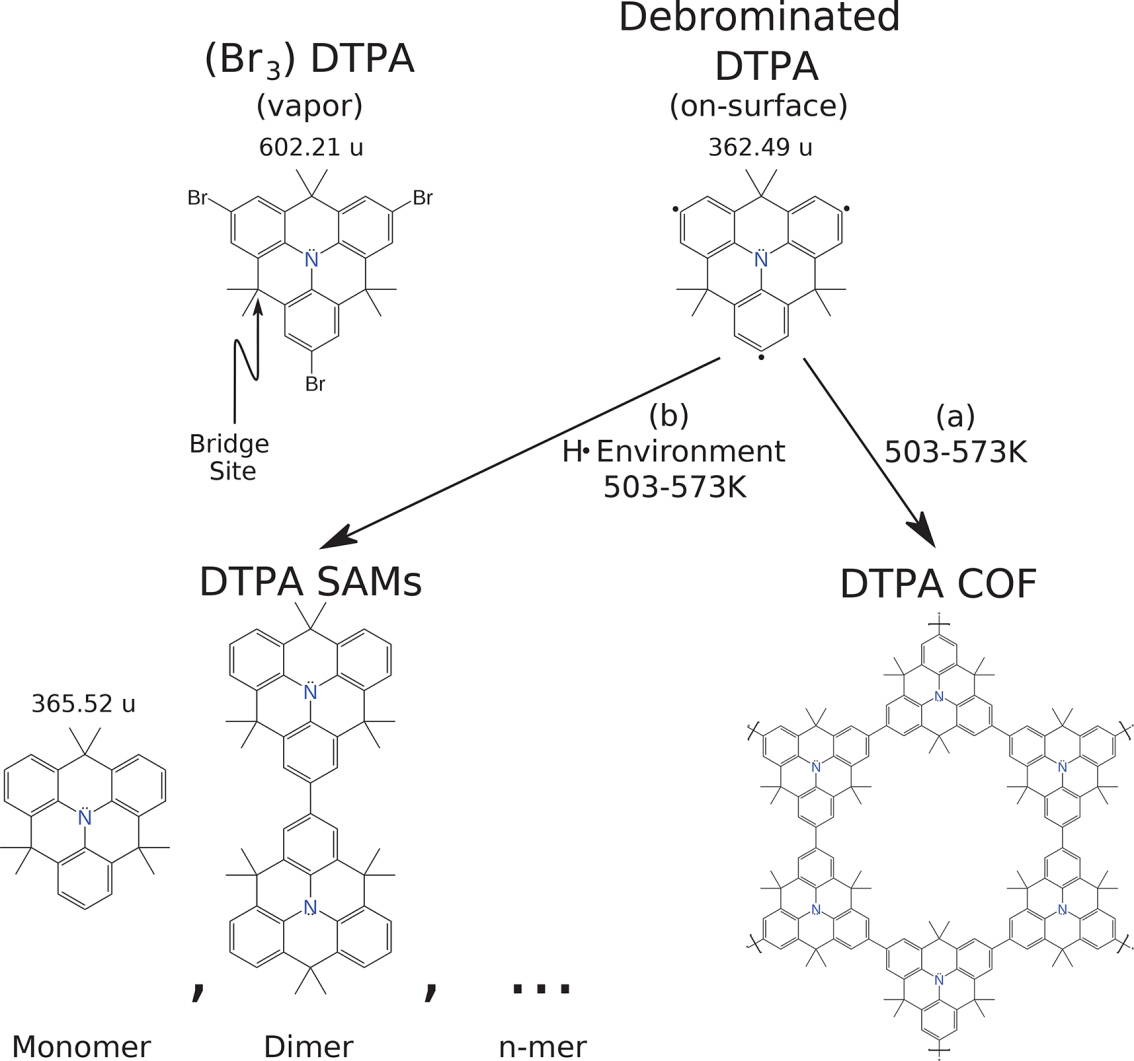
On-surface reaction of tribromo-DTPA precursor molecules on a heated metallic substrate in an atomic hydrogen-rich or high-vacuum environment. (Br_3_)DTPA molecules are evaporated onto a Ag(111) substrate held at a constant temperature within 503 to 573 K. First reaction step: Dehalogenation of (Br_3_)DTPA resulting in surface-stabilized DTPA radicals. Second reaction step: Bonding at debrominated sites, dependent on the environmental conditions: (a) completion of Ullmann-type coupling forming C–C bonds between DTPA molecules or (b) reaction with atomic hydrogen forming C–H bonds on a fraction of the radical sites. This terminates the polymer at *n* units, with *n* determined by kinetics.

The monomer precursor used in this work and its proposed on-surface radical configuration are shown in Figure 1, as well as the observed products for (a) UHV deposition and (b) deposition in the presence of atomic hydrogen. In these experiments, (Br_3_)DTPA precursor molecules were deposited from heated crystallized monomers onto temperature-controlled Ag(111) substrates. When deposited on a surface, the methyl groups point perpendicular to the surface and dominate the STM imaging, forming the triangular shapes for the molecules. An important point is that experiments were done in a multiuser STM facility; therefore, to limit potential contamination of the main UHV chambers, depositions were conducted in the turbo-pumped sample-introduction chamber (load-lock) with an ambient pressure of (2–3) × 10^–8^ mbar (HV). These conditions produced results which are cautionary in a sense, yet promise an additional level of control over on-surface synthesis.

**Figure 2:**
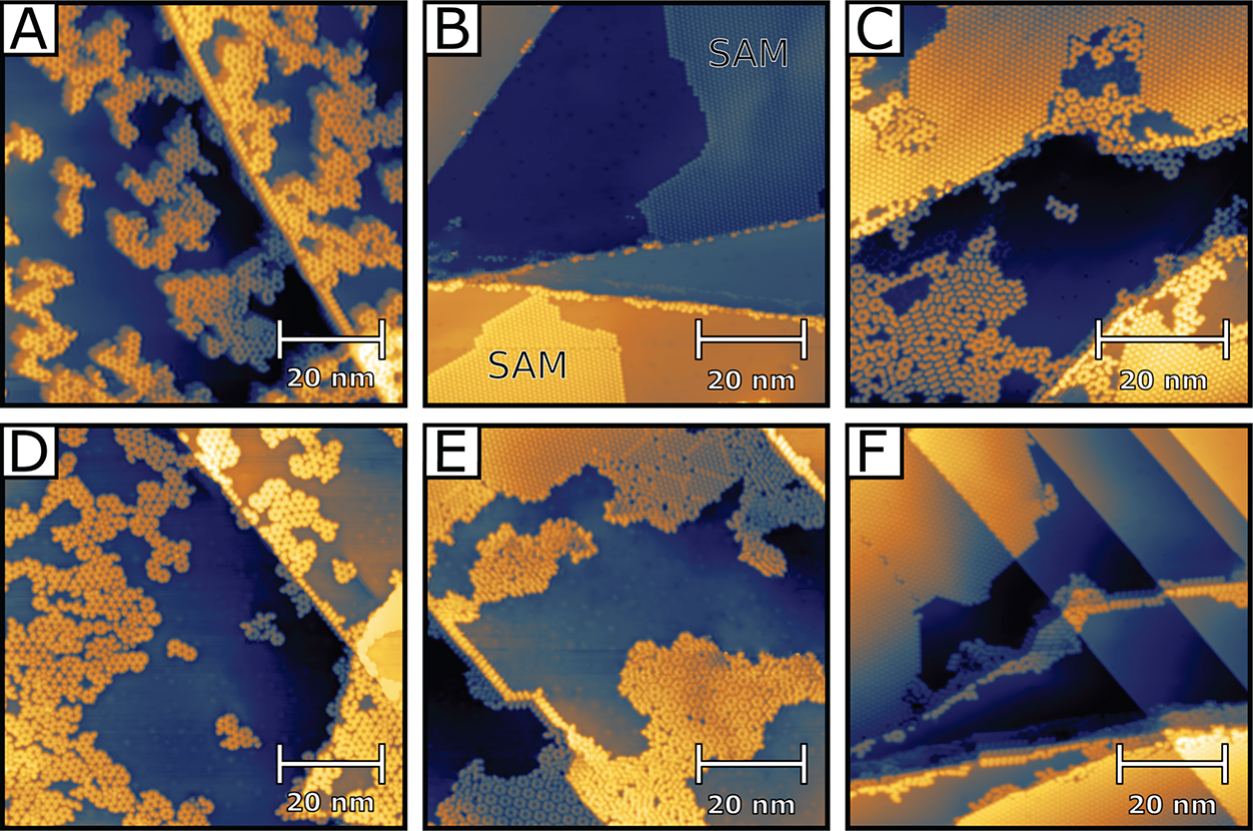
STM topographs of DTPA depositions under various environmental conditions. All depositions are on a Ag(111) substrate at temperatures of 523 K (A–C) and 503 K (D–F) with a deposition rate of 0.055 ML/min (A–C) and 0.025 ML/min (D–F). (A–C) Depositions in a chamber backfilled with H_2_ to 2.2 × 10^–6^ mbar with (A) the cracking filament off throughout the process, (B) the cracking filament on (0.1 mA emission current) during DTPA deposition, and (C) the clean Ag(111) sample in the chamber with the cracking filament on (0.1 mA emission current) for 10 min prior to a DTPA deposition with the filament off. (D–F) Depositions in an HV chamber (*P* = 2.9 × 10^–8^ mbar) with (D) the cracking filament off throughout the process, (E) the cracking filament on with 0.1 mA emission current, and (F) the cracking filament on with 1 mA emission current (for more information, see Methods).

Initial deposition attempts showed that the ((Br_3_)DTPA) covalent bonding process on Ag(111) was inhibited, a result that was traced to the on/off state of a remote (no line-of-sight path to the silver substrate) thoriated-iridium filament in the load-lock ion gauge (see Supporting Information Figure S1). Adopting the hypothesis that in HV this filament acts as a “cracking” source (H_2_ → 2H^•^), inhibiting aryl–aryl bonding through competitive C–H bonds, we conducted the experiments summarized in Figure 2. These depositions were conducted with varying amounts of environmental atomic hydrogen, controlled by changing the amount of molecular hydrogen in the chamber or the filament temperature (by increasing the emission current setting of the ion gauge). During deposition, the substrate was held at a constant temperature, from 503 to 523 K, chosen within the lower end of the range indicated in Figure 1 because the hydrogen-terminated DTPA monomers were found to desorb from the surface at higher temperatures (see Supporting Information Figure S2). The primary finding is seen by the different concentrations of DTPA structures present between Figure 2A and B. Figure 2A is the control deposition with the cracking filament off throughout the time the sample is in the deposition chamber (before, during, and after deposition) and in the presence of additional H_2_ backfilled to a pressure of 2.2 × 10^–6^ mbar. The observed 10 to 20 nm COF islands are consistent with prior results,^8,12^ and no other significant structures are observed. In contrast to this, Figure 2B shows a deposition conducted in the same elevated molecular hydrogen environment and the cracking filament turned on only during DTPA deposition. In this case, only large islands of monomer self-assembled monolayers (SAMs) are observed. An additional experiment presented in Figure 2C found an unexpected result. The image in Figure 2C was acquired after pre-exposing the sample to 2.2 × 10^–6^ mbar H_2_ gas with the cracking filament on for 10 min prior to deposition, turning off the filament, pumping the H_2_ from the chamber, and then performing the DTPA deposition. This resulted in a mixture of structures showing more oligomer SAM formations than the control deposition in Figure 2A but also more covalent coupling than Figure 2B.

The results shown in the top row of Figure 2 support the hypothesis that it is atomic hydrogen, produced at the remote cracking filament, which inhibits on-surface C–C bond formation and hence COF formation. We confirmed that the source of atomic hydrogen is thermal cracking of H_2_ at the hot filament, rather than energetic electrons or ions produced by accelerating potentials in the ion gauge (see Figure S3). Due to its lower operating temperature (∼1700 K at 0.1 mA emission), thoriated iridium has a lower cracking efficiency than tungsten operating at the same emission,^30^ yet clearly produces sufficient H^•^ to dramatically influence on-surface reactions. Furthermore, the results from Figure 2C show that the Ag(111) surface (or possibly other nearby surfaces) serves as a reservoir for H^•^. Once filled by pre-exposure to a source of atomic hydrogen, this reservoir allows H^•^ to affect the on-surface reaction long after the cracking filament is off and the H_2_ has been evacuated.

Other depositions, in HV conditions and with the cracking filament on, show that the fractions of COF and SAMs (monomer, dimer, hexamer) which form depend sensitively on the deposition rate, the substrate temperature (Figure S2), and the H^•^ partial pressure. An example of this is seen by the controlled depositions shown in Figure 2D–F. Each deposition uses the same deposition rate, substrate temperature, and background H_2_ pressure but varies the temperature of the cracking filament by changing its emission current (see Methods). As the filament temperature increases, its H_2_ cracking efficiency increases, resulting in higher H^•^ production. The increase in H^•^ causes greater disruption in the DTPA covalent coupling which is seen by the progression from COF islands in Figure 2D, to a mixture of oligomer SAMs in Figure 2E, and finally to predominantly monomer SAMs in Figure 2F (see also Figures S4–S7). This is consistent with two chemical species (debrominated DTPA and atomic H) competing to bond to the same radical sites. However, quantitative confirmation for any of the reaction products proposed in Figure 1 cannot be determined from STM alone.

**Figure 3:**
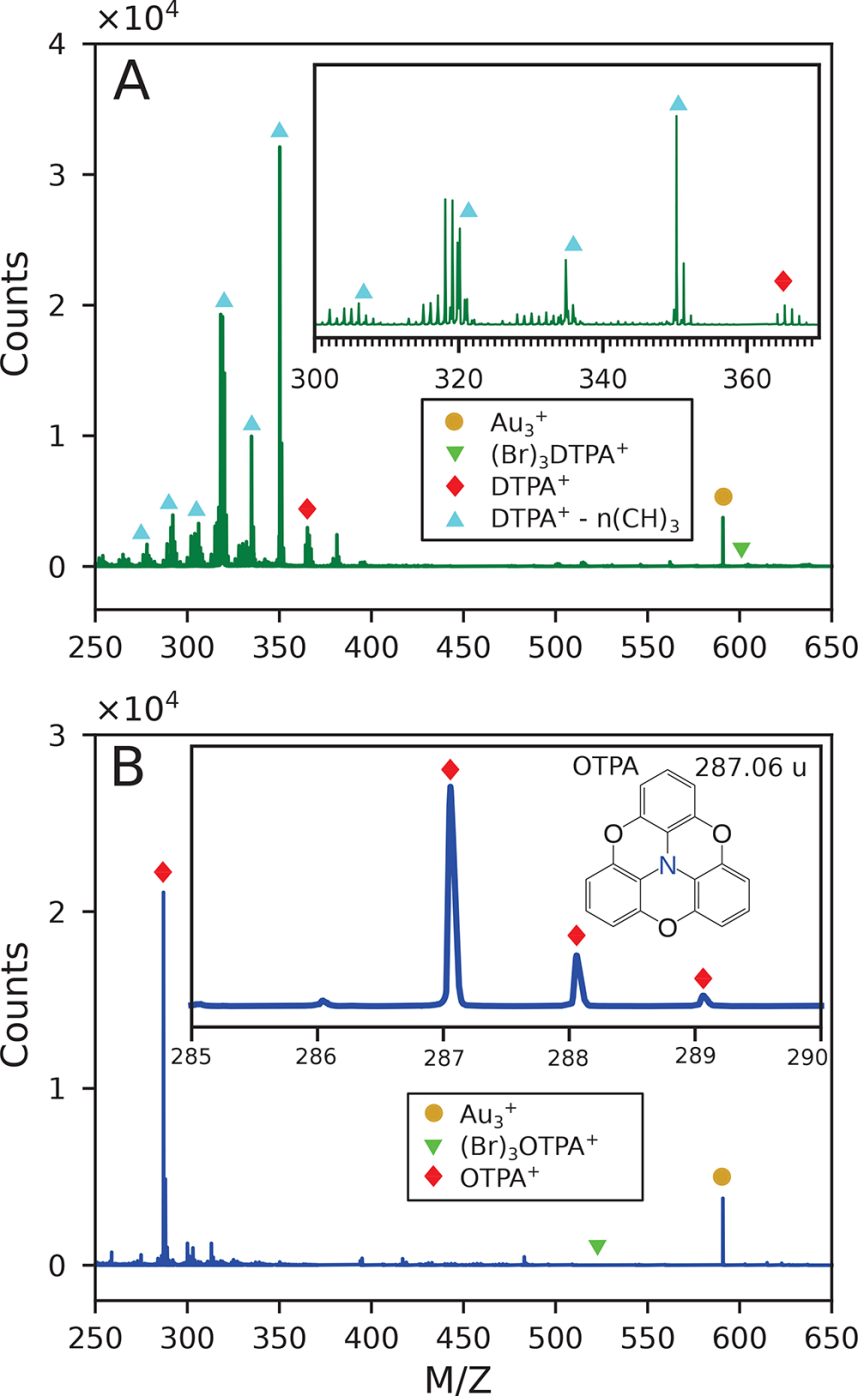
Positive ion TOF-SIMS analysis of a monolayer of (A) DTPA monomer SAM and (B) OTPA monomer SAM. The layer was obtained by deposition of the brominated precursors onto a heated Au(111) surface in the presence of a cracking filament in an environment of 2.2 × 10^–6^ mbar H_2_. The DTPA SAM shows fragmentation characterized by groups of peaks separated by roughly 15 u, which suggests the cleavage of bridging methyl groups due to the energetic secondary ion generation process. In contrast, the OTPA spectrum shows only one prominent set of peaks, due to the absence of methyl substituents. The peak at 287.06 u in (B) corresponds to OTPA^+^, with smaller side peaks due to its natural isotopic mass distribution.

To confidently identify the on-surface reaction products, we turn to TOF-SIMS. Monomer DTPA samples similar to the one shown in Figure 2B were prepared on Au(111), imaged by STM (Figure S8), and then transferred through the atmosphere to the TOF-SIMS instrument. The samples were prepared on Au(111) instead of Ag(111) to avoid surface contamination by oxygen. Figure 3A displays the relevant portion of the mass spectrum, with the inset expanding the mass scale near the monomer DTPA^+^ (cf. Figure 1). A key observation is vanishingly small intensities at masses corresponding to (Br_3_)DTPA^+^ or any other partially brominated DTPA species. For masses smaller than monomer-DTPA^+^, the spectrum shows fragmentation due to the cleavage of increasing numbers of bridging methyl groups, resulting in evenly spaced peak sets, as denoted by light blue triangles in Figure 3A. Within the peak set encompassing the exact mass (red diamonds), all peaks are well-described by natural isotopic abundances, with the largest peak at 365.5 u corresponding to the fully hydrogenated DTPA^+^. However, the more intense peak sets at lower masses are further complicated by metastable states, which have intensity at fractional masses due to in-flight transformations.^31^ Despite these complications, the mass spectrum of prepared monomer SAMs indicates that the monomers are, as hypothesized, simply hydrogen-terminated DTPA molecules. That is, H has replaced Br in the (Br_3_)DTPA precursor molecules (see also Figure S9).

Still, the complexity of the mass spectrum in Figure 3A may leave some doubt as to the robustness of this conclusion. To provide further support, we performed an analogous experiment on monomer SAMs of the related oxygen-bridged molecule OTPA,^32^ shown in the inset to Figure 3B (precursors (Br_3_)OTPA, deposition conditions similar to those of Figure 2B on Au(111); see also Figure S10). Without the bridging methyl groups, the cracking pattern seen in mass spectra from OTPA samples (Figure 3B) is far simpler. A single prominent hydrogen-terminated OTPA^+^ mass peak is found at the expected mass of 287.06 u, with associated minor peaks due to the natural isotopic abundances of the constituent atoms. Also, like mass spectra for DTPA, there is virtually zero intensity corresponding to any brominated species. This provides further support that H has replaced Br in the monomer product. Repeating the experiment using deuterium instead of hydrogen as a background gas shows the presence of OTPA terminated with deuterium instead of hydrogen, providing further confirmation of the proposed process (see Figure S11 and accompanying discussion). The results of the deuterium experiment also confirm that the hydrogenation (and deuteration) occurs inside the vacuum chamber and not during transfer of the sample to the TOF-SIMS instrument (∼5 min exposure to the atmosphere).

### Controlling On-Surface Synthesis

**Figure 4:**
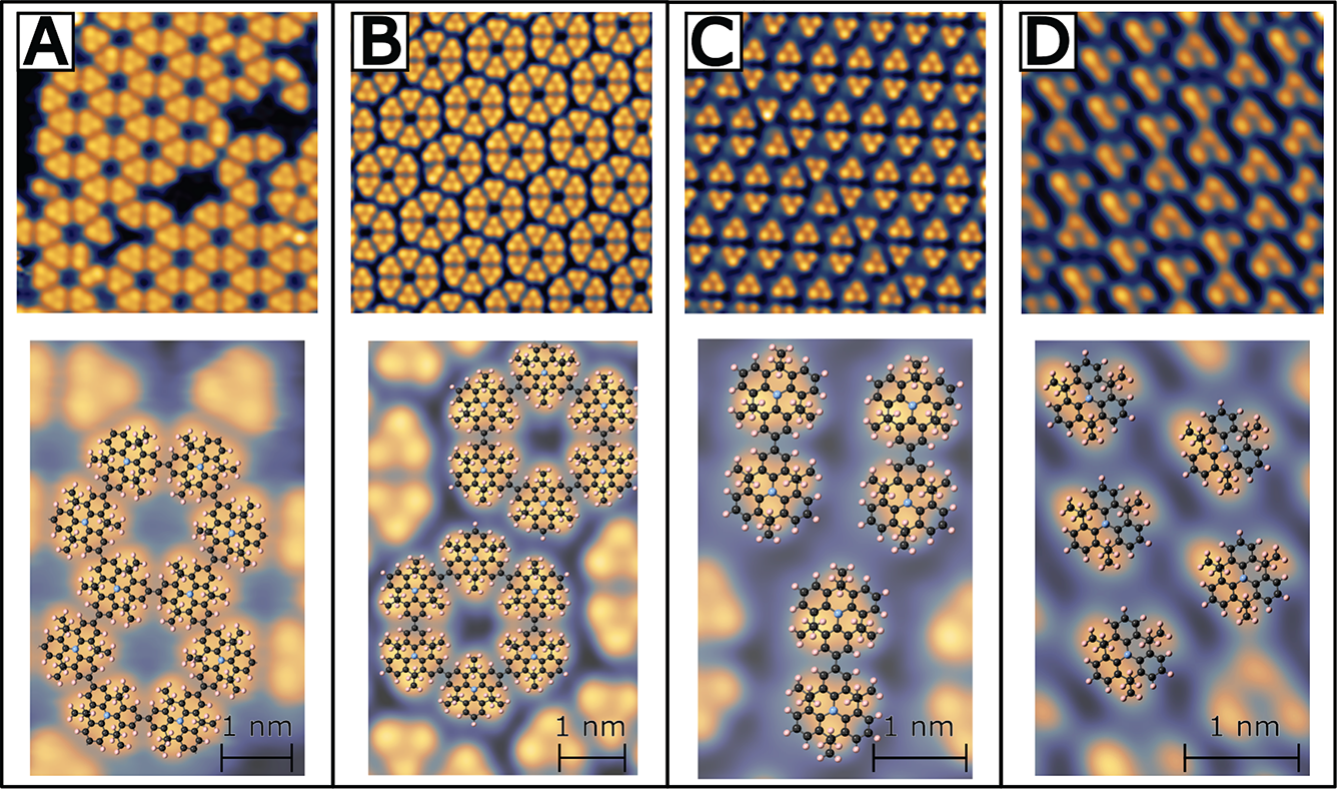
Experimental STM topographs of DTPA SAMs and COF superimposed with models. (A) DTPA COF. (B) Hexamer SAM. (C) Dimer/trimer SAM. (D) Monomer SAM. First row: experimental topographs; second row: enlarged topograph superimposed with structural model (hydrogen-terminated DTPA for SAMs). The DTPA SAM images were acquired at 77 K, while the COF data were acquired at 4.7 K. (For specific deposition and imaging parameters, see Methods.)

As established above, the presence of atomic hydrogen during on-surface synthesis inhibits the Ullmann reaction between organic molecules. Now we show that, by controlling deposition parameters, this effect can be used to synthesize a useful sequence of oligomers from the same (Br_3_)DTPA precursors. Figure 4 shows the variety of compounds and their SAMs formed by deposition in controlled reducing environments. These topographs were chosen to emphasize the characteristic molecular structures and their assembly, not the entire surface distribution of oligomers/COF, which depends on the deposition parameters and is statistical in nature, as shown in Figure 2 and further quantified below.

Clearly, the molecular lattices of the SAMs vary for different oligomers, yet they share some similarities. Our first observation is simply the formation of SAMs, which implies that the intermolecular attractions are sufficiently strong to overcome the repulsive electric dipoles caused by charge transfer between adsorbed molecules and the silver substrate.^33−36^ Second, from the molecular models depicted in the bottom row of Figure 4, the minimum spacing between oligomer edge C atoms is greater than 0.42 nm in all cases, so the attractive interaction is likely to be dominated by dispersion forces.^37^ Finally, we find that the molecular lattices have preferential orientations with respect to the substrate and appear to be commensurate with the Ag(111) lattice (see discussion of Figures S12 and S13 in the Supporting Information).

Because hydrogen is equally likely to bind to any radical site on the DTPA molecule, the synthesis of different oligomers is statistical in nature. A different flux of atomic hydrogen during DTPA deposition results in a different distribution of oligomers on the surface. We have investigated the statistics of oligomer SAMs and COF from three depositions made with identical parameters, except for the cracking filament temperature, which is set by its emission current (Figure 2D–F). As shown in Table 1, we find that the fraction of self-assembled monomers increases as the filament temperature (i.e., cracking rate or H^•^ flux) increases, while the percentage of molecules in the hexamer self-assembly decreases. The effectiveness of the reducing environment can be quantified as the number of newly bound H atoms per DTPA, deduced as three minus the number of aryl–aryl bonds per monomer, as shown in Table 1 (see also Supporting Information, Figures S4–S7).

**Table 1:** Distribution of Oligomer Species on the Ag(111) Substrate for Different Cracking-Filament Emission Currents^a^

emission (mA)	area (nm^2^)	no. molec.	1-mer %	2-mer %	6-mer %	H/DTPA
0.04	153 500	31 500	13	15	20	1.7
0.10	126 300	32 200	36	4	16	2.0
1.00	226 400	45 500	67	8	0	2.5

a Fractions of molecules in monomer (1-mer), dimer (2-mer), and hexamer (6-mer) SAMs are shown, as well as the total area imaged and the number of molecules. The average number of hydrogen terminations per DTPA molecule (H/DTPA) is calculated considering all molecules, including those in mixed regions of oligomers and partial-COF that do not form well-ordered self-assembly.

Table 1 shows that the oligomer concentrations on the surface can be dramatically changed by the introduction of atomic hydrogen during deposition. Thus, the ability to create this reducing environment using nearly universal tools on vacuum systems could be of interest for future studies. However, the statistical data and images presented (here and in the Supporting Information) also show that the formation kinetics of the oligomers is complex. For instance, from imaging we find that hexamer SAMs tend to occur within regions of monomer SAMs and that desorption of monomers becomes highly probable at temperatures required for aryl–aryl bonding. Table 1 also exposes some of this complexity: the fraction of dimers decreases and then increases as the environment becomes more reducing (higher emission). A deeper understanding of the kinetics would enable more detailed control of the on-surface chemistry.

### Electronic Structure Evolution

Direct comparison between on-surface experiments and the theoretical electronic structure of free-standing COFs can be challenging due to a potentially strong interaction between the metal substrate and the COF adlayer. As implied by Figure 4 (read from D to A), we can view the COF as the end point of a progression of molecular structures. We propose that the on-surface synthesis of these increasingly complex oligomers could lead to a more complete experimental understanding of the on-surface COF electronic structure.

**Figure 5:**
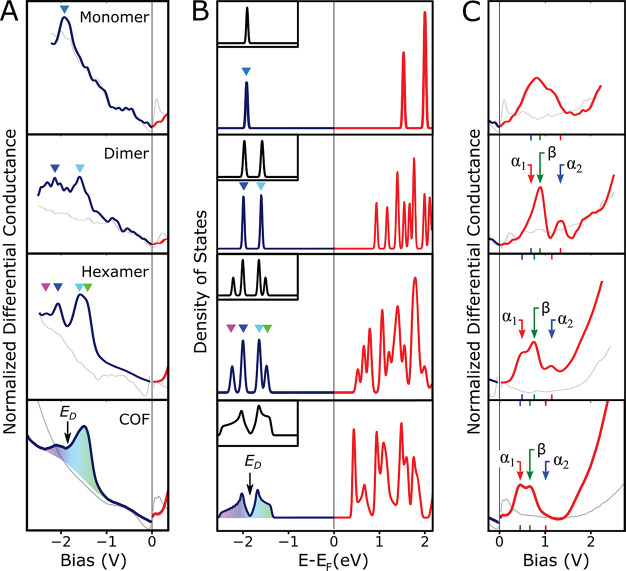
Comparison of the progression of occupied levels (blue) and unoccupied levels (red) from monomer to 2D COF. (A) Normalized differential conductance spectra ((d*I*/d*V*)/(*I*/*V*)) taken via STS over monomer SAM, dimer SAM, hexamer macrocycle SAM, and DTPA COF (top to bottom) on Ag(111). Blue lines are spectra over the molecular structure, and gray lines are reference spectra taken with the same tip over bare Ag(111) at the same tunnel gap impedance. The monomer and dimer spectra are individual measurements representative of a larger data set, while the hexamer and COF spectra are averages obtained from spectral maps. (B) Freestanding DFT calculations of the occupied DoS (blue) and unoccupied DoS (red) for the monomer, dimer, and hexamer molecules and the DTPA COF (top to bottom). The Fermi levels were set by aligning the calculated HOMOs with experimental spectra in (A). Inset boxes are single-orbital tight binding calculations plotted on the same energy axis, with the tight binding parameters *t*_*nn*_ = −0.2 eV and *t*_*nnn*_ = −0.012 eV, where *t*_*nn*_ and *t*_*nnn*_ are the nearest-neighbor and next-nearest-neighbor hopping elements. (C) Continuation of the normalized differential conductance spectra from (A) but over the unoccupied electronic states. Labels α_1_, α_2_, and β mark peaks with similar spatial distributions, as shown in Figure 6. The SAM data were acquired at 77 K, while the COF data were acquired at 4.7 K.

Figure 5A (Figure 5C) displays experimental filled-state (unfilled-state) STS obtained within SAMs of monomers, dimers, hexamer macrocycles, and a COF island. In Figure 5B we show the density-of-states (DoS) calculated at the DFT-PBE level for the freestanding oligomers and COF. For the highest occupied molecular orbital (HOMO) levels, both experiment and theory show a simple trend of peak splittings, which is consistent with the intuitive notion of a linear combination of molecular orbitals (LCMO): one MO contributed by each monomer.^38^ A simplified effective model of interacting MOs can be implemented using a tight-binding model with a single orbital per site^7^ (see Figure S14 and accompanying text). The insets to Figure 5B show that this model reproduces the basic STS and DFT results, including the symmetry-expected 1:2:2:1 degeneracies of the hexamer DoS peaks and the slight asymmetry in the peak energies for the hexamer and COF bands (within the model, the asymmetry is due to next-nearest-neighbor interactions).

Over the same energy range as the hexamer HOMO levels, the calculated COF spectrum (bottom panel of Figure 5B) shows similar spectral characteristics, except that individual levels broaden into bands. Experimentally, the correspondence is less obvious due to the energy-dependent background in STS (light gray lines show STS over the silver surface). The amplitude and width of STS features may also be affected by the finite size of the COF island, lattice defects, and energy-dependent mechanisms such as the hole lifetime. However, tracking the progression of electronic levels to their COF end point enables an unambiguous experimental identification of the HOMO bands and particularly the Dirac point (*E*_D_) for these bands, which clearly lies at the minimum STS intensity near −1.9 eV (see also the band structures calculated at the DFT-PBE level in Figure S15). In Figure 5B, the DFT spectra have been shifted to align with the experimental spectra by matching *E*_D_ and other prominent features.

In contrast to the filled states, the evolution of the lowest unoccupied molecular orbitals (LUMOs) is complicated from the outset by the presence of closely spaced MOs in the monomer, as shown in the density functional theory (DFT) results of Figure 5B. Effective tight-binding models using a basis set of π_*x*_, π_*y*_, and σ orbitals have previously illuminated interesting aspects of 2D COF band structure,^38−40^ but such an approach is less intuitive for this system with several closely spaced MOs. More importantly, the experimental spectra from unfilled electronic states (Figure 5C) bear little resemblance even to our DFT results. We take this as an indication that—over the LUMO energy range—the influence of the substrate is significant, which is undoubtedly due to the presence of the Ag(111) surface state above −55 meV. Deciphering the observed spectral peaks in this situation is difficult, but it is facilitated by having regular arrays of successively larger oligomers, where the influence of the surface state can be isolated to some extent.

Spectra taken over the SAMs and COF consistently show distinct local maxima within the range 0 to 1.5 eV. The peak energies, widths, and amplitudes vary from monomer through hexamer, but become similar for the hexamer and COF. For discussion, α_1_, β, α_2_, and accompanying lines/tics in Figure 5C mark peaks of potentially similar origin, as determined by their spatial distribution.

**Figure 6:**
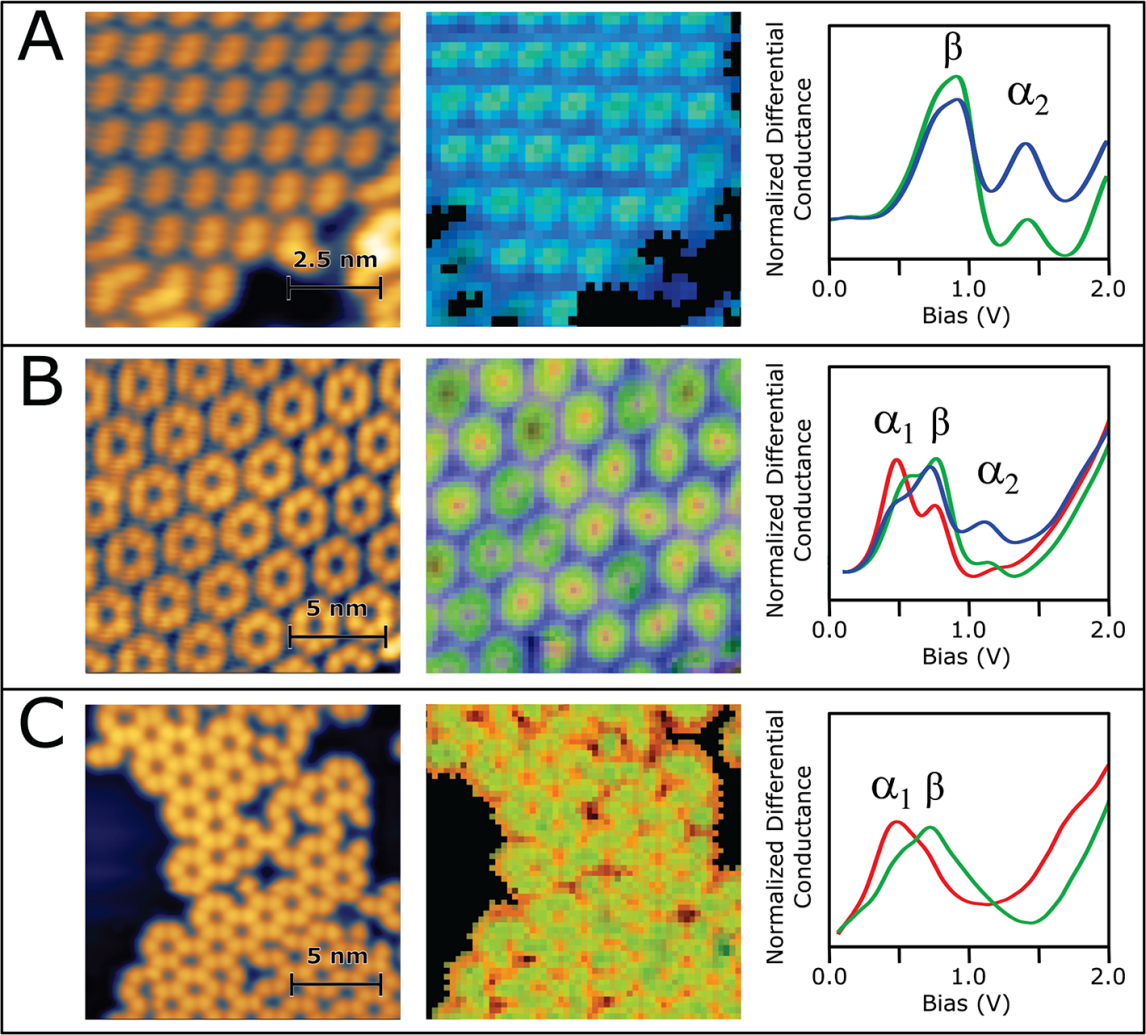
Each row shows the results of spectral maps taken over a dimer SAM (A), hexamer SAM (B), and DTPA COF (C). In each row the first image is the STM topograph. For images in the middle column, RGB colors show the spatial distribution of characteristic “origin” spectra plotted in the third column, i.e., spectra from green-colored portions of the maps are similar in character to the green spectrum, etc. Characteristic spectra were determined as averages over K-means clusters (see Figure S16). Similarity is quantified as the normalized Euclidean distance (2-norm, computed in the vector space spanned by the bias voltages) of a point spectrum from a chosen characteristic spectrum. Similarity maps in the color of each characteristic spectrum (with the most intense color for smallest Euclidean distance) were overlaid to produce the composite maps shown. Figure S16 The dimer SAM and COF spectral maps were acquired at 4.7 K, while the hexamer data were acquired at 77 K.

The left column of Figure 6 displays topographs taken over regions of dimer SAM (A), hexamer SAM (B), and COF (C), with data from corresponding spectral maps shown in the middle and right columns. The K-means clustering algorithm^41^ was used to group 64 × 64 spectra from each map into four (dimers and COF) or five (hexamers) clusters, based on their spectral features within 0 to 2 V (see Figure S16 for additional details). Of these clusters, one always corresponds to spectra from the Ag(111) substrate and another to spectra displaying transient “tip-switches” or other anomalies; these two clusters are not considered in further analysis. Spectra within each of the remaining clusters (labeled and colored as red, green, or blue) are averaged and shown in the right column of Figure 6 (these average spectra comprise the “origin vector” to the center of each cluster, where the size of the vector space is the number of bias-voltage samples). Comparing with Figure 5C, it is clear that the K-means unsupervised machine learning picks out spectra that are dominated by different peaks among α_1_ (red), β (green), and α_2_ (blue). We visualize the distribution of each cluster (roughly speaking, each spectral peak) over the imaged regions using color intensity to represent the normalized Euclidean distance (vector 2-norm) of individual spectra from their respective cluster centers. The resulting images are shown in the middle column of Figure 6, with different cluster data represented by intensities on different color channels of the same image (colors correspond to spectra in the right column).

The red-cluster spectra from the hexamer SAM and COF have α_1_ as the tallest peak and are located predominantly over the pores. This is inconsistent with the calculated charge density maps for the freestanding COF LUMO (see Figure S17) or, indeed, any COF level. Peak β is present in all cluster spectra but has the highest relative intensity in the green cluster, which overlies the molecular backbone for all three structures (dimer SAM, hexamer SAM, and COF). Finally, the α_2_ peak attains its highest intensity in the blue-cluster spectra which are found in the interstitial regions of the SAMs. The α_2_ peak essentially vanishes for the COF, which has no interstitial region, and is also absent for an isolated hexamer spectrum, a further indication that it relates to the SAM formations.

Comparing the spatial distribution of spectral data from the COF and different SAMs facilitates the separation of features dominated by molecular states versus those dominated by the substrate surface state. With their prevalence over regions of bare substrate, we associate the red and blue K-means clusters with metal-dominated features (α_1_ and α_2_ peaks), whereas the green cluster corresponds to molecule-dominant LUMO states. “Quantum corral” confinement of noble-metal surface states has been known for many years^42^ and has more recently been studied and modeled for metal–organic frameworks and organic SAMs.^43−46^ A straightforward calculation for a circular quantum well of radius 0.94 nm (the COF pore diameter) results in a confinement energy of 0.54 eV (see Supporting Information discussion “Electron confined to a circle in two dimensions”). This is consistent with the energy of peak α_1_ for both the hexamer and the COF, so we have some confidence that its source is resonant scattering of the Ag(111) surface state within the pores. Similarly, the spectral maps of Figure 6 show that peak α_2_ is largely a consequence of a surface state resonance in the interstitial regions of the dimer and hexamer SAMs, for which the intermolecular spacings are approximately the same. This conclusion is supported by a plane-wave model of surface-state electrons propagating in a 2D lattice of model Gaussian potentials, each situated under an atom of the polymer (see Figure S18 and accompanying text). The model predicts a resonance at around 1.0 eV, which is close to the observed energy of α_2_. Finally, we note that the absence of peak α_2_ for the COF spectrum of Figure 5 is expected in this interpretation since no interstitial region exists. We interpret a weak shoulder near 1 eV as a second-order pore resonance, which is also reproduced in these scattering models.

Peak β may also be influenced by the metal surface state, but its spatial distribution implies that it derives mainly from the adsorbed molecular layer. Identifying β as the LUMO peak, we can extract a more accurate bandgap than otherwise possible. For our COF experiments, the derived value of the bandgap, 1.85 ± 0.10 eV, is nearly identical to DFT results for the freestanding COF (Figure 5B). (This close agreement may be fortuitous; we expect that the calculated bandgap would decrease somewhat with the inclusion of a Ag(111) substrate beneath the COF.)

## Conclusions

This work establishes the disruptive effect of atomic hydrogen during the on-surface synthesis of covalent organic frameworks, with quantitative confirmation of the hydrogenated products that impede Ullmann coupling. We find that even a remote cracking filament and modest hydrogen pressure can create a sufficiently reducing environment to dramatically inhibit C–C bonding in ambient high vacuum and postulate that in ultrahigh vacuum the same effect could limit the size and quality of 2D COF crystals. Therefore, to achieve large-area 2D COFs in vacuum, the level of background hydrogen must be minimized, and bare filaments should be cold during COF synthesis. These findings will affect the design of heating stages, chamber construction, and pump selection for dedicated 2D COF facilities. Our research also shows that these inhibitory processes can be controlled and exploited to produce—from a single monomer precursor—a progression of oligomers (monomers, dimers, hexamers, and to a lesser extent, 3-,4-,5-mers) organized into self-assembled monolayers, which have their own utility and scientific interest. Other 2D organic networks, with analogous oligomer sequences, could benefit from the deposition and analysis techniques demonstrated here. Finally, we expect that deposition in this controlled reducing environment can be used to manage the length distribution of surface-synthesized one-dimensional polymers, ensuring a known hydrogen termination and allowing the exploration of length-dependent properties such as molecular conductance^47^ and electronic structure.^48^

## Methods

### Precursor Synthesis and Depositions

(Br_3_)DTPA and (Br_3_)OTPA precursor molecules were synthesized as per the reported procedures.^49−51^ DTPA depositions were conducted in the baked-out load-lock with a pressure of (2–3) × 10^–8^ mbar. DTPA precursor molecules are evaporated from a home-built Knudsen dual-crucible evaporator, made of boron nitride, onto a UHV sputter-cleaned Ag(111) substrate approximately 0.4 m away. The Ag(111) thin films, grown on cleaved mica,^52^ were prepared by cycles of sputtering and annealing in ultrahigh vacuum (UHV) prior to each deposition. The substrate is held at a constant temperature during deposition and for 5–10 min after the deposition is complete. The sample is then moved into the UHV chamber (<1 × 10^–10^ mbar) and onto the cooled STM stage. Depositions in the H^•^-rich environment have 99.9999% purity H_2_ continuously streamed into the load-lock chamber via an attached variable leak valve, maintaining a specified pressure. Depositions with the “cracking source on” were performed with a Bayard–Alpert ion gauge equipped with a thoriated iridium filament operating with a hot filament emission current of 0.04, 0.1, or 1 mA measured at the grid generated by passing 4.0, 4.1, and 4.4 A, respectively, through the filament. The temperature of the filament is maintained by the ion gauge controller using the emission current as feedback.

Deposition parameters vary for Figure 4. Figure 4A had the ion gauge filament off throughout the duration the sample was in the load-lock. The background pressure was less than 2.3 × 10^–8^ mbar for the 12 min deposition at a deposition rate of 0.05 ML/min (molecular monolayers per minute) onto a 300 °C Ag(111) substrate. For Figure 4B, the sample was held in the load-lock chamber for 15 min with the ion gauge on and a background pressure of 2.4 × 10^–8^ mbar prior to the deposition. The deposition was then conducted with the ion gauge off for 11 min at a deposition rate of 0.05 ML/min onto a 300 °C Ag(111) substrate. Figure 4C exposed the sample to the ion gauge for 20 min prior to deposition in the load-lock with a background pressure of 4.7 × 10^–8^ mbar. The ion gauge was left on during the 8.25 min deposition at a rate of 0.065 ML/min onto a 315 °C Ag(111) substrate. Finally, Figure 4D had the sample exposed to the ion gauge for 30 min prior to deposition in the load-lock with a background pressure of 2.8 × 10^–8^ mbar. The ion gauge remained on during the 10.75 min deposition at a rate of 0.045 ML/min onto a 300 °C Ag(111) substrate.

The densities of the monomer and dimer assemblies are 7.4 × 10^13^ molecules per cm^2^, while the densities of the hexamer assembly and COF are 8.6 × 10^13^ and 7.6 × 10^13^ molecules per cm^2^, respectively.

### STM

Measurements were performed with a CreaTec LT-STM operating in UHV (<1 × 10^–10^ mbar) at 77 K. All STM images were taken in constant current mode with W tips etched in the lab and cleaned via *in situ* field emission over a clean Ag(111) substrate and subsequent conditioning, typically via nanoscale contact with the Ag substrate. All STM conductance spectra presented were acquired as part of 64 × 64 spectroscopic maps, where d*I*/d*V* spectra are acquired after opening the feedback loop at each grid point. The STM operates in constant current mode between spectra. A typical acquisition time for a topograph is 30 s, and the specific tunneling conditions for each image are listed below. We used Gwyddion^53^ to process images (e.g., line leveling, color range, Gaussian filter). Tunneling spectra were analyzed by a home-built Python tool. d*I*/d*V* spectra are normalized by taking (d*I*/d*V*)/(*I*/*V*), where *I* is generated by numerically integrating a heavily smoothed (d*I*/d*V*) signal, and data points very close to the Fermi level (zero sample bias) are removed. d*I*/d*V* data are obtained using the internal digital lock-in amplifier integrated into the CreaTec hardware/software, which directly modulates the bias voltage and demodulates the current signal. In this work the lock-in frequency is 1111 Hz and the lock-in amplitude is 45 mVpp.

The topographs in Figure 2 were acquired at (sample bias, tunnel current) (A) −1.95 V, 58 pA; (B) 2.07 V, 63 pA; (C) 1.80 V, 87 pA; (D) −1.13 V, 69 pA; (E) −0.85 V, 120 pA; (F) 1.35 V, 16 pA.

The topographs in Figure 4 were acquired at (A) – 0.28 V, 150 pA; (B) −0.43 V, 96 pA; (C) −1.83 V, 63 pA; (D) −2.03 V, 120 pA.

The spectral map data presented in Figure 6 were acquired in an open loop mode with the tunneling set points (A) 0.56 V, 220 pA; (B) −0.50 V, 120 pA; (C) −0.52 V, 26 pA.

### TOF-SIMS

Samples were prepared by *in situ* deposition of monomers onto a Au(111) thin film substrate to a uniform coverage of 1 ML as determined by STM. A characterized sample was then transferred to a TOF-SIMS instrument (IONTOF 5-300), requiring under 10 min  exposure to the atmosphere. After transfer and subsequent vacuum pump-down, the samples were analyzed using a primary ion beam of Bi_3_^2+^ cluster ions with an energy of 50 keV and a secondary ion extraction voltage of 2 kV. The cumulative spectra shown in Figure 3 were acquired during raster scans of the primary ion beam over 500 μm × 500 μm regions of the samples.

### Density Functional Theory

DFT calculations were performed with the projector augmented wave (PAW) method,^54^ as implemented in the Vienna Ab initio Simulation Package (VASP).^55,56^ The generalized gradient approximation (GGA)/Perdew–Burke–Ernzerhof (PBE) functional^57^ including the Grimme dispersion correction (DFT-D3)^58^ was used for geometry optimizations and electronic structure calculations. In the geometry optimization of the 2CH_3_-bridged triphenylamine COF monolayer, the cutoff energy was set to 500 eV and the Γ-centered 9 × 9 × 1 *k*-grids with Monkhorst–Pack scheme were adopted. Both the lattice parameters and atomic coordinates were fully relaxed until the force on each atom and the variation in total energy were smaller than 0.01 eV Å^–1^ and 1 × 10^–6^ eV, respectively. For the single-point electronic DoS calculations, a 21 × 21 × 1 *k*-mesh was used. In the geometry optimizations of the DTPA monomer, dimer, and hexamer, the same convergence criteria as the COF monolayer was adopted using only the Γ point. For the interface system of the CH_2_-bridged triphenylamine COF on a five-layer Ag slab, the bottom three Ag layers were fixed, while the top two Ag layers and the COF were fully relaxed. K-meshes of 3 × 3 × 1 and 9 × 9 × 1 were used for the geometry optimizations and electronic structure calculations, respectively.

## Supporting Information Available

Geometry of load-lock deposition chamber, STM images of DTPA deposition at different substrate temperatures, experimental data demonstrating that the hot filament in the ion gauge is the primary cracking source, experimental data showing the varying surface composition of DTPA species with varying cracking filament temperature, STM images of DTPA deposition on Au(111) substrate, additional ToF-SIMS data with Br-terminated and H-terminated DTPA, STM images of OTPA deposition on Au(111) substrate, ToF-SIMS data obtained from deuterium-substituted OTPA, description of the commensurate molecular DTPA overlayers on Ag(111) surface, DFT calculations of the adsorption configurations on Ag(111) surface, description of the tight binding calculations, band structures of freestanding DTPA COF obtained by DFT, additional description of the STS spectral map analysis in Figure 6, partial charge density maps of the freestanding DTPA COF obtained by DFT, description of electron confined in a pore, and description of the electron plane wave expansion modeling (PDF)
