# Supplementary material for: Tailoring On-Surface Molecular Reactions and Assembly through Hydrogen-Modified Synthesis: From Triarylamine Monomer to 2D Covalent Organic Framework
Source: ACS Nano. 2023 Apr 4;17(8):7366–76. doi: 10.1021/acsnano.2c11463 (PMC10134737; doi:10.1021/acsnano.2c11463)
Supplement: Supplementary file 1 — nn2c11463_si_001.pdf [file nn2c11463_si_001.pdf]

Supporting Information

for

"Tailoring On-Surface Molecular Reactions and Assembly through Hydrogen-Modified Synthesis: From Triarylamine Monomer to 2D Covalent Organic Framework"

Zachery A. Enderson,† Harshavardhan Murali,† Raghunath R. Dasari,‡ Qingqing Dai,¶ Hong Li,¶ Timothy C. Parker,‡ Jean-Luc Brédas,¶ Seth R. Marder,‡,§,‖ and Phillip N. First*,†

†*School of Physics, Georgia Institute of Technology, Atlanta, GA 30332, United States*

‡*School of Chemistry and Biochemistry, Georgia Institute of Technology, Atlanta, GA 30332, United States*

¶*Department of Chemistry and Biochemistry, The University of Arizona, Tucson, AZ 85721, United States*

§*University of Colorado Boulder, Renewable and Sustainable Energy Institute, Department of Chemical and Biological Engineering, Department of Chemistry, and Materials Science and Engineering Program, Boulder CO 80303, United States*

‖*National Renewable Energy Laboratory, Chemistry and Nanoscience Center, Golden CO 80401, United States*

E-mail: first@gatech.edu

Load-lock Deposition Geometry

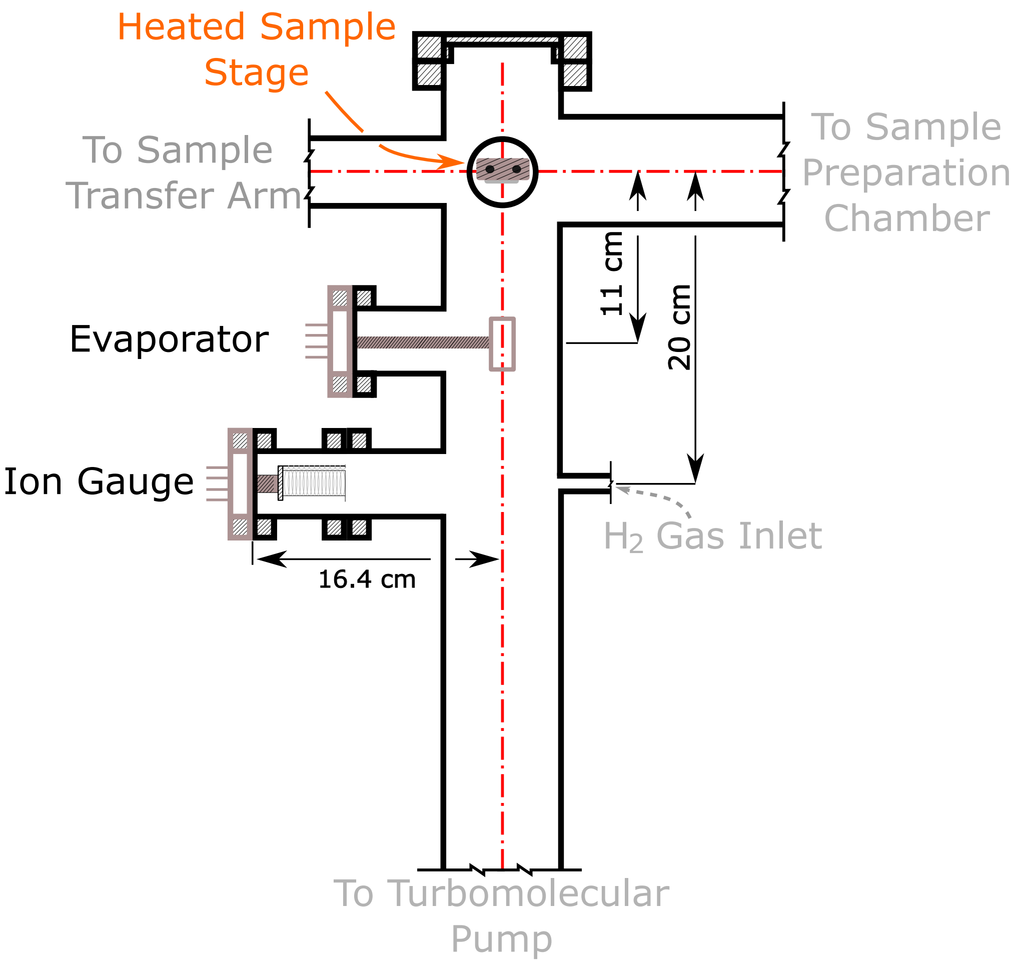

Figure S1: Schematic of the turbo-pumped sample-introduction chamber (load-lock) used for molecular depositions. Molecules originate from the dual crucible evaporator positioned 11 cm from the heated sample stage. The H_2 cracking filament is the primary filament on the ion gauge positioned below the sample stage and evaporator.

Substrate Temperature and Monomer Desorption

Desorption of the hydrogen terminated DTPA monomers was found to occur at lower substrate temperatures than for other oligomers (Figure S2). The desorption was observed by a decrease in molecular coverage after depositing in a H_2 backfilled environment with the cracking filament on (which leads to approximate total hydrogenation of the debrominated

DTPA sites). At 543 K substrate temperature no molecules are present on the surface (Figure S2A), whereas the control deposition (filament off) has ≈ 70% monolayer coverage (B). At 523 K substrate temperature the filament-on deposition has ≈ 32% monolayer coverage (C) whereas the control deposition had ≈ 65% monolayer coverage (D).

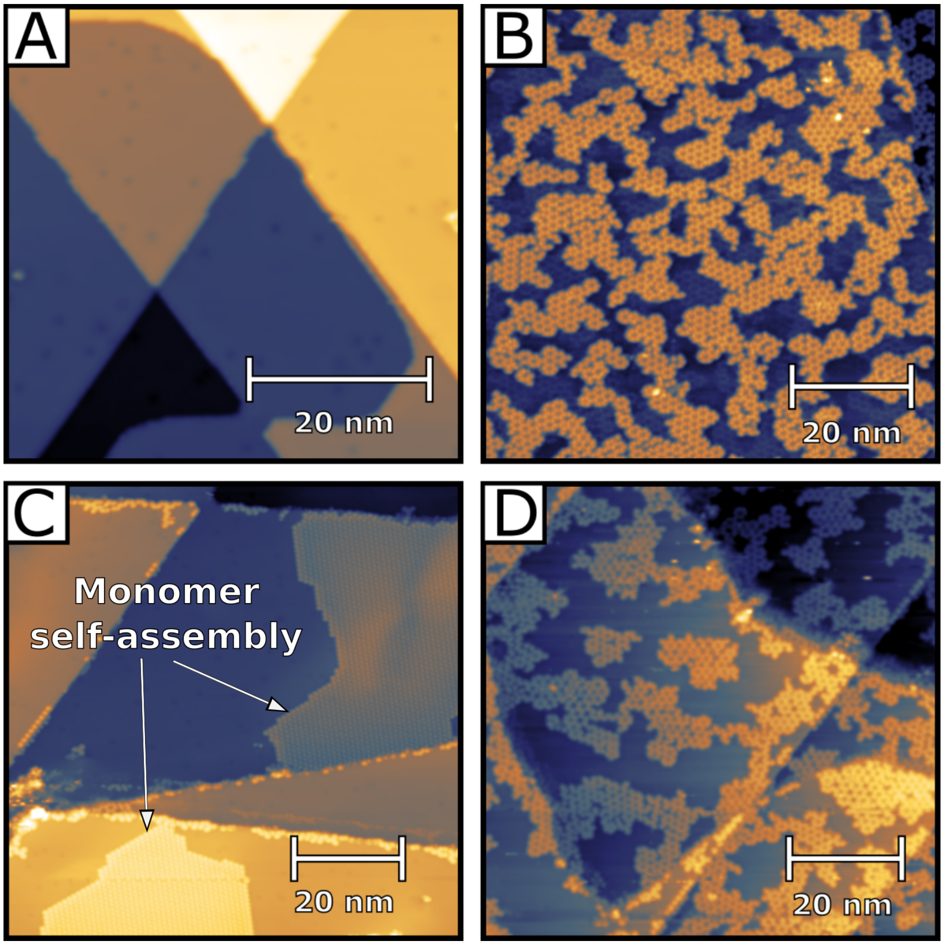

Figure S2: Topographs of DTPA depositions under different conditions on Ag(111). (A) H_2 backfilled to 2.2×10^{-6} mbar with cracking filament on (0.1 mA emission current) and 543 K substrate temp. (B) HV deposition with cracking filament off and 543 K substrate temp. (C) H_2 backfilled to 2.2×10^{-6} mbar with cracking filament on (0.1 mA emission current) and 523 K substrate temp. (D) HV deposition with cracking filament off and 523 K substrate temp. Imaging conditions: (A) 1.82 V, 24 pA, (B) −2.07 V, 29 pA, (C) 2.07 V, 63 pA and (D) −1.80 V, 140 pA.

Verification of the hot filament as the primary source of atomic hydrogen

To verify that the most significant source of H• is the heated filament of the ion gauge and not the generated ions or electrons, Figure S3 shows a deposition of DTPA in the presence of H_2 with only a heated ion gauge filament (no grid or filament bias voltages). This resulted in a surface covered with only monomer SAM; the same result as obtained by deposition in H_2 with the ion gauge operating normally (i.e., with grid and filament bias voltages).

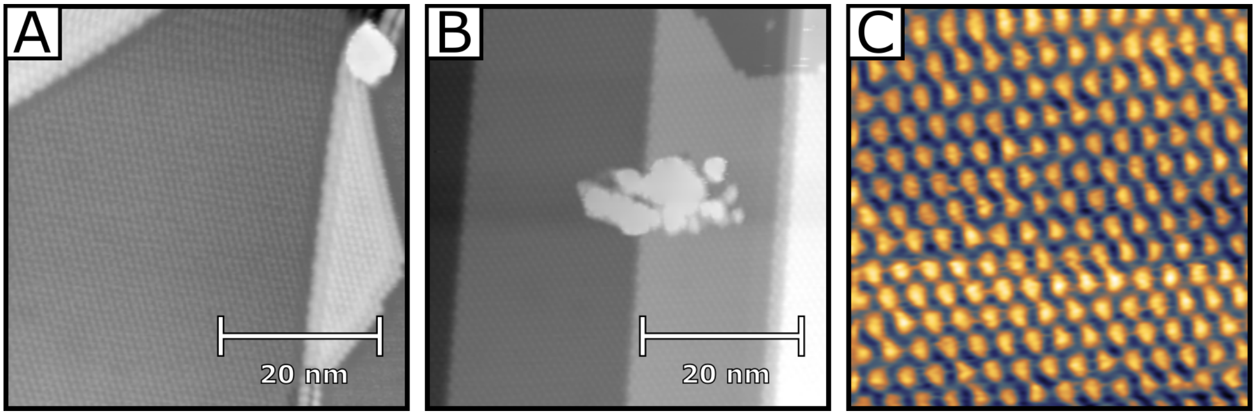

Figure S3: STM topographs of a DTPA deposition onto Ag(111) performed in a H_2 backfilled chamber (2.2×10^{-6} mbar) and the heated ion gauge filament. The filament was heated directly by an AC current of 4.15 A (approximate filament current for 0.1 mA emission current) without a grid voltage. This deposition showed only large (50 nm to 100 nm) islands of monomer SAMs. (A,B) Examples of large monomer SAMs. (C) Zoom of monomer SAM region. Imaging parameters: (A,B) 1.18 V, 28 pA and (C) −1.18 V, 22 pA.

DTPA on Ag(111): Surface composition with varying cracking filament temperature

The deposition of (Br_3)DTPA onto a heated Ag(111) substrate in the presence of a source of atomic hydrogen results in a mixture of DTPA species with varying degrees of hydrogenation, due to the stochastic nature of the termination due to atomic hydrogen. When a hot filament is absent, the surface shows the formation of covalently-bonded islands even in the presence of molecular hydrogen as shown in Figure S2B,D.

Performing the depositions with an excess of atomic hydrogen results in virtually all the molecules being fully hydrogenated, as the surface is covered only by the monomer SAM, for example, Figure S2C, S3C. Depositions with lower amounts of hydrogen produce mixtures of oligomers. A fraction of these various oligomeric species form well-ordered self-assemblies.

To illustrate this, we have performed three depositions with identical molecular deposition rates, background pressures (3×10^{-8} mbar) and substrate temperatures (503 K), for the same duration of time (10 minutes) while varying the temperature of the cracking filament. The temperature of the cracking filament was varied by changing the thermionic emission current of the filament. Depositions were performed with emission currents of 0.04 mA, 0.1 mA and 1 mA, and the surfaces were scanned using STM to identify the differences in the sample composition.

The species found on the surface can be broadly categorized as self-assembled monomers (Figure S4A), dimers (B), hexamers (C), and mixed oligomers (D).

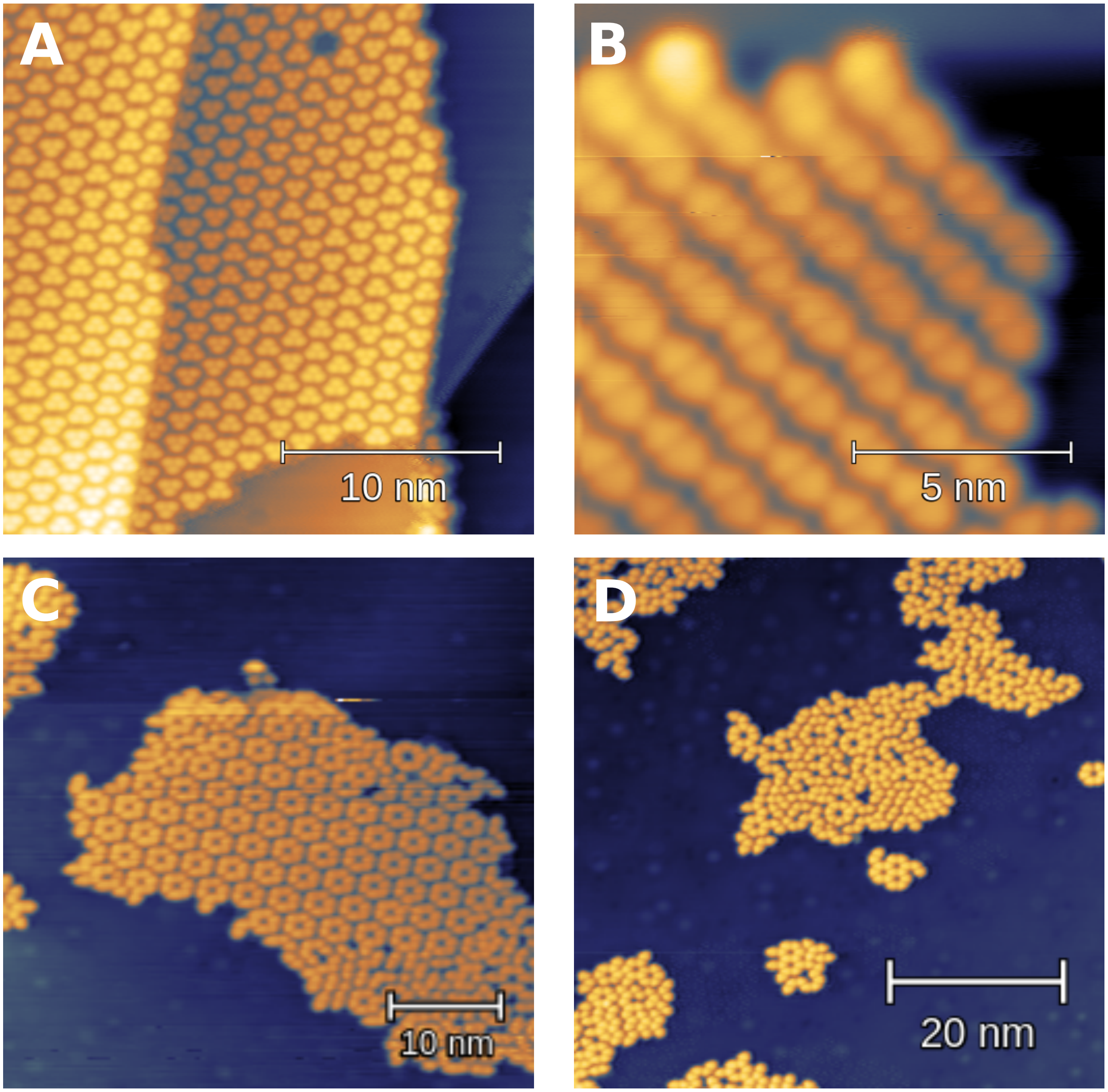

Figure S4: Species typically found on the Ag(111) surface after deposition of DTPA in the presence of atomic hydrogen. The commonly found ordered self-assemblies are that of (A) monomers, (B) dimers and (C) hexamers. In addition to the ordered self-assemblies, aggregates of oligomers without well-defined self-assembly (D) can also be found. Imaging parameters: (A) −0.97 V, 130 pA (B) −1.44 V, 25 pA (C) −0.86 V, 110 pA and (D) −2.00 V, 150 pA.

The results of the depositions broadly follow the expected trend of higher filament temperature causing a greater degree of hydrogenation of the DTPA species.

1 mA emission: This results in a surface that is dominated by monomer self-assembly, along with a small proportion of dimer self-assembly and mixed oligomers (Figure S5). Hexamer self-assembly was not seen when scanning this sample.

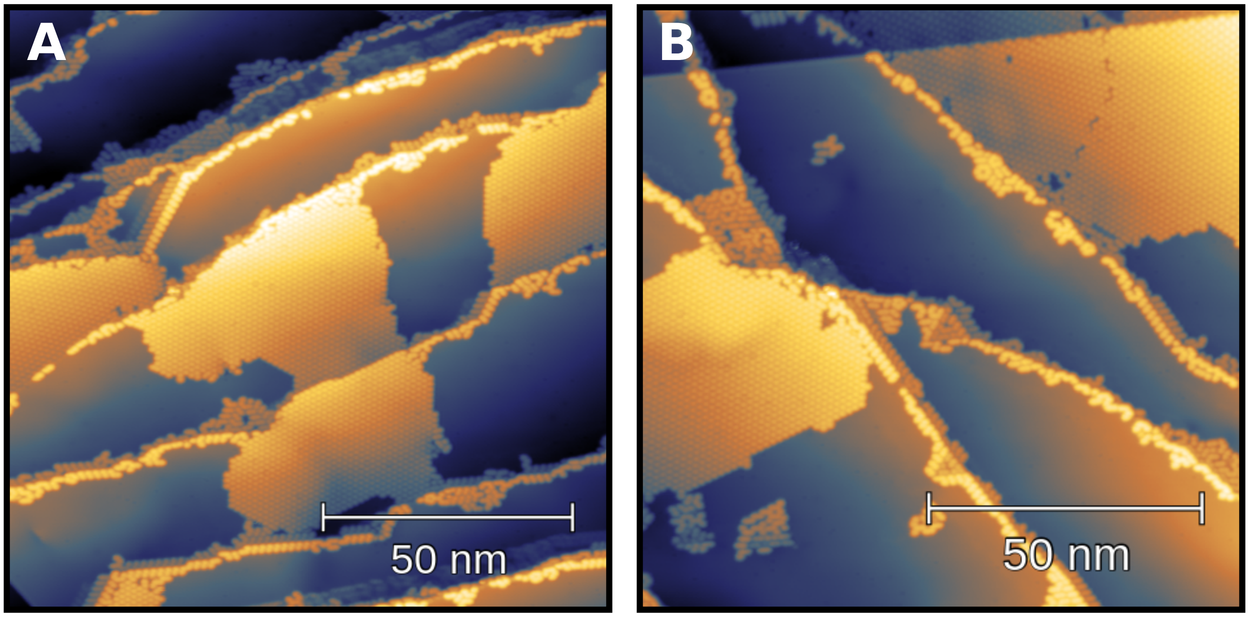

Figure S5: Deposition performed with a filament emission current of 1 mA. The surface is dominated by monomer self-assembly, with small islands of dimers and mixed oligomers on the surface and step-edges. Imaging parameters (for both (A) and (B)): 1.35 V, 16 pA.

0.1 mA emission: In this setting, ordered self-assemblies of monomer, dimers and hexamers, as well as mixed assemblies of oligomers were found (Figure S6). The monomer assemblies are mostly intact, with some disruption by dimers, etc. (Figure S6A).

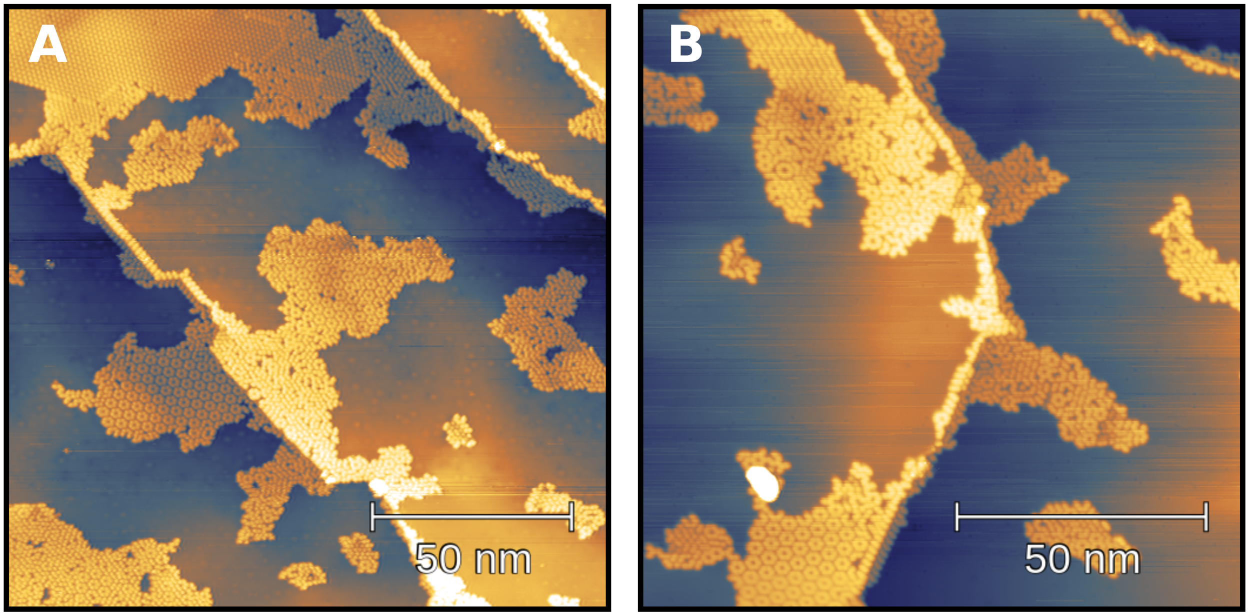

Figure S6: Deposition performed with a filament emission current of 0.1 mA. The surface contains self-assembled monomers, dimers, hexamers as well as oligomers. Imaging parameters: (A) −0.85 V, 120 pA and (B) 1.13 V, 32 pA.

0.04 mA emission: The surface is similar to 0.1 mA emission but with a larger amount of hexamer self-assembly and with a larger portion of the monomer self-assemblies disrupted by rows of dimers or disordered assemblies of a mixture of species.

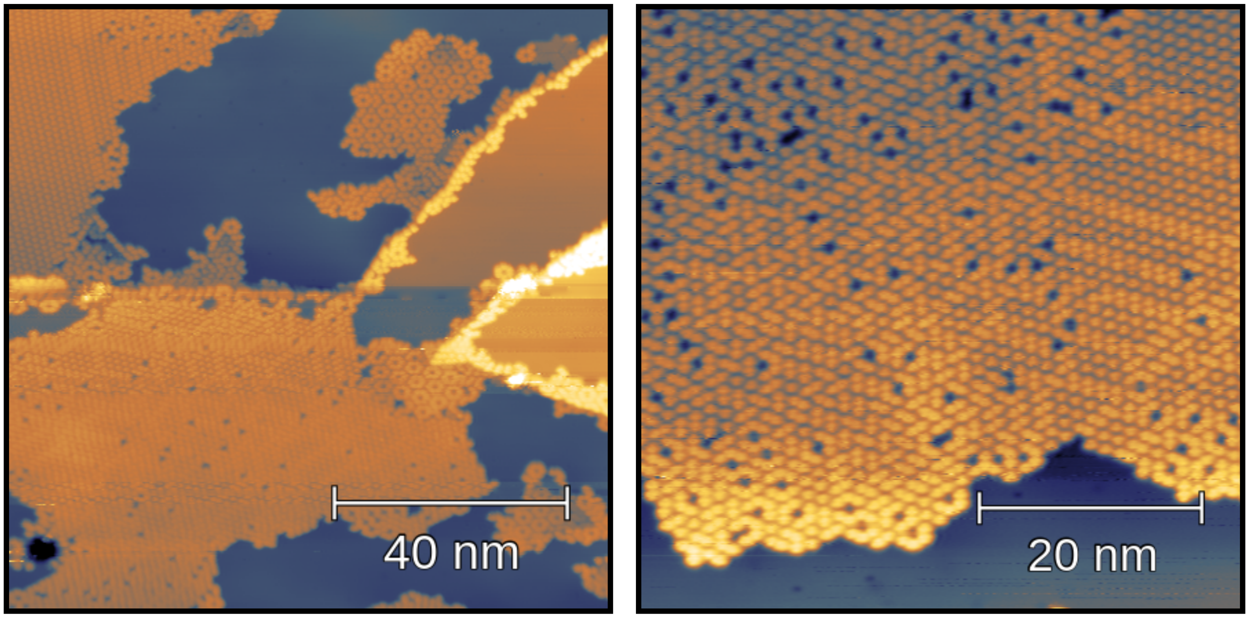

Figure S7: Deposition performed with a filament emission current of 0.04 mA. The surface looks similar to the results obtained with 0.1 mA. However, larger areas of the monomer self-assemblies are disrupted with regions containing a mixture of dimers, trimers and other oligomers (B). Imaging parameters: 1.59 V, 44 pA.

The results of the STM data obtained from these three samples is summarized in Table 1 of the main text, and reproduced for convenience in Table S1 below. In order to estimate the average number of hydrogen atoms per DTPA molecule in the Table, we assume that every DTPA termination site not bonded to another DTPA molecule is instead bonded with one hydrogen atom. Hence, each DTPA monomer has 3 hydrogen atoms, each dimer has 4 terminal H atoms (two per DTPA molecule), etc. Then, we look at select sample regions with mixed oligomers, as well as the disrupted monomer self-assembly (see, e.g., Figure S7B) and obtain an estimate of the average number of DTPA-H bonds per DTPA molecule and an average area density of the DTPA molecules by manually counting the bond order. Finally, using the area densities of the various self-assemblies, we estimate the number of molecules in the scans by manually highlighting the area covered by each of the mentioned categories

and estimate the average degree of hydrogenation of the molecules.

Table S1: Distribution of oligomer species on the Ag(111) substrate for different cracking-filament emission currents. Fractions of molecules in monomer (1-mer), dimer (2-mer), and hexamer (6-mer) SAMs are shown, as well as the total area imaged and the number of molecules. The average number of hydrogen terminations per DTPA molecule (H/DTPA) is calculated considering all molecules, including those in mixed regions of oligomers and partial-COF that do not form well-ordered self-assembly.

Emission	Area	No. molec.	1-mer	2-mer	6-mer	H/DTPA
0.04 mA	153 500 nm^2	31 500	13%	15%	20%	1.7
0.10	126 300	32 200	36	4	16	2.0
1.00	226 400	45 500	67	8	0	2.5

DTPA Depositions on Au(111)

Depositions prepared for TOF-SIMS experiments were performed on Au(111) substrates rather than the Ag(111) substrate used throughout the text. This was done to avoid potential oxidation of the substrate during transfer to the mass spectrometer. Figure S8 shows the results of the DTPA experiments performed on a Au(111) substrate. Similar to the Ag(111) substrate, the molecules form two dimensional networks when deposited in the absence of a source of atomic hydrogen, while they form a self-assembled structure without bonding when the deposition is performed in the presence of atomic hydrogen.

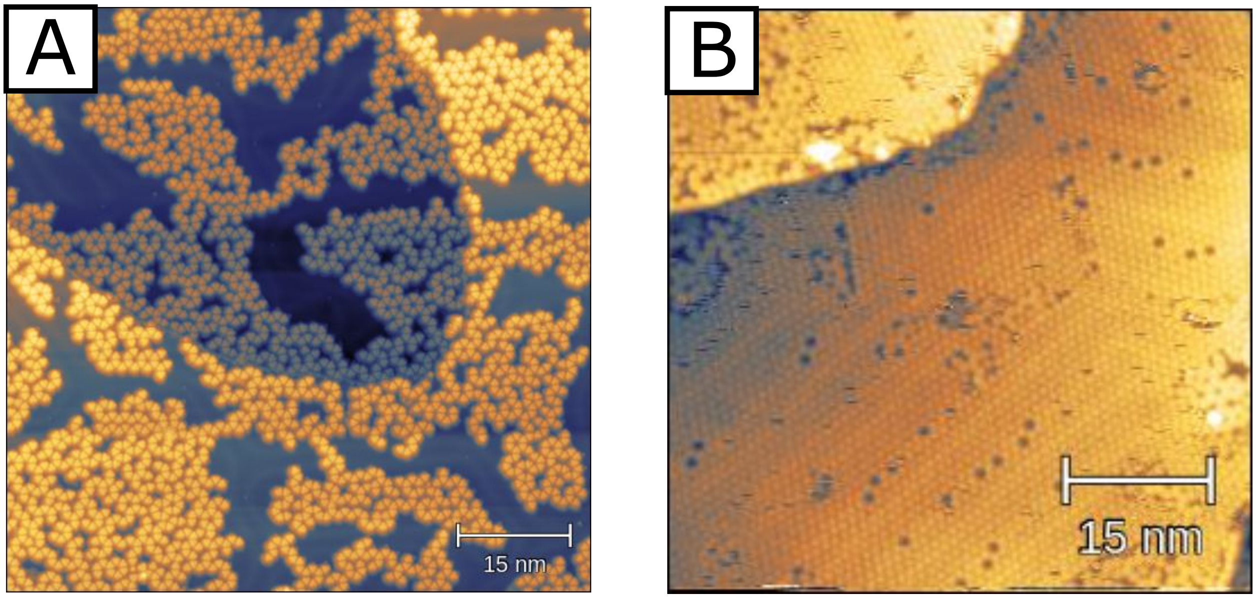

Figure S8: DTPA Molecules deposited on Au(111) substrate in the (A) absence of atomic hydrogen, and (B) in the presence of 2.2×10^{-6} mbar of hydrogen in the chamber along with a cracking filament. The depositions were performed at a temperature of 523 K(est.)*. Imaging parameters: (A) -1.09 V, 90 pA and (B) 1.78 V, 21 pA.

*Faulty thermal contact made temperature measurements of the Au(111) substrate unreliable, so temperatures were judged by applied power. Temperatures for Figures S8-S10 were affected. Results of subsequent experiments are consistent with the estimated temperatures.

ToF-SIMS: Br-terminated and H-terminated DTPA

In this section, we provide additional data for the quantitative evidence of hydrogenation of the molecules. Figure S9 shows the mass spectra of the direct deposition of (Br_3)DTPA on the Au(111) substrate at room temperature without any atomic hydrogen (red) and deposition on a hot substrate in the presence of atomic hydrogen (green). The peaks of the non-hydrogenated sample are located 3u lower than the corresponding peaks of the hydrogenated sample. The reduction in mass by 3u indicates that the peaks are caused by fragments of (Br_3)DTPA that lose the Br atoms in the ion generation process, but do not have hydrogen on the three termini.

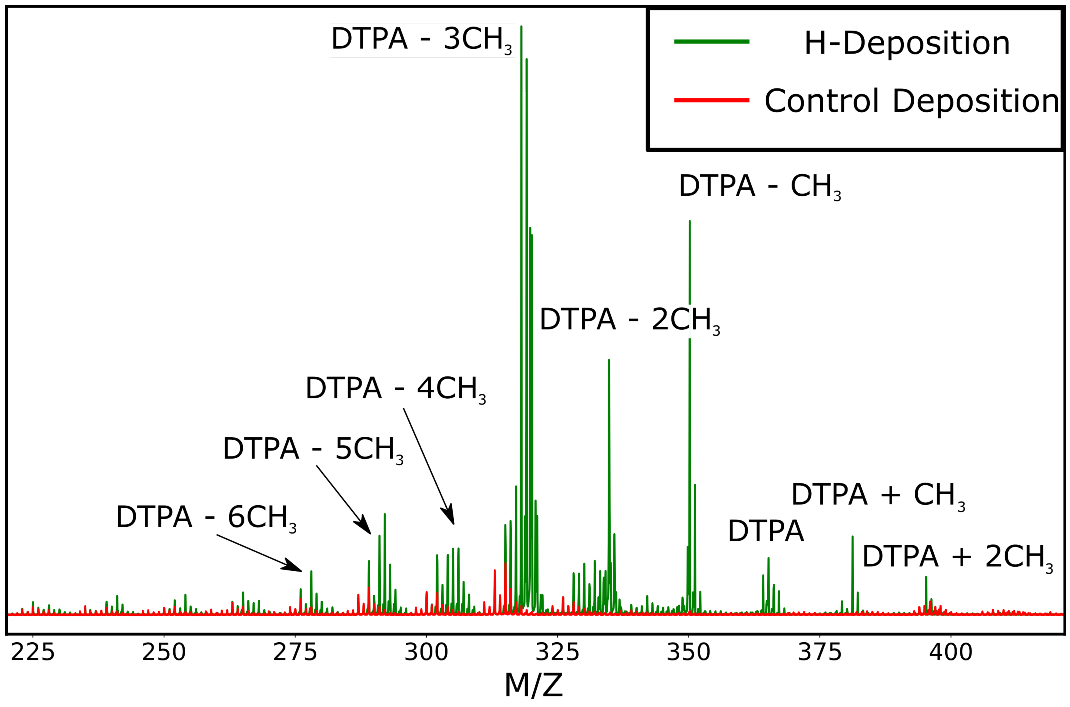

Figure S9: Selection of the ToF-SIMS mass spectrometry data for the non-brominated DTPA mass ranges. The green spectrum is the experimental data for DTPA molecules deposited in a H_2-rich environment with the cracking filament on. The molecules were deposited onto a Au(111) sample heated to 523 K(est.). The red spectrum is a control deposition of DTPA molecules onto a room temperature Au(111) sample without the presence of additional H_2 and the cracking filament off.

OTPA Depositions on Au(111)

Figure S10 shows the results of the deposition of OTPA on the Au(111) surface. These molecules form a two dimensional covalent network when deposited without any sources of atomic hydrogen (A), and form a self-assembled monolayer when deposited in the presence of atomic hydrogen (B). The OTPA molecules image differently in STM as compared to the DTPA molecule; the absence of the nearly vertical methyl bridging groups means that the STM images the OTPA molecules with the brominated sites at the vertices of the triangles(as opposed to the DTPA methyl bridge sites as the vertices). The self-assembled phase we observe is similar to the un-annealed, self-assembled phase of the (Br_3)OTPA molecule on Au(111) reported in the literature.[1]

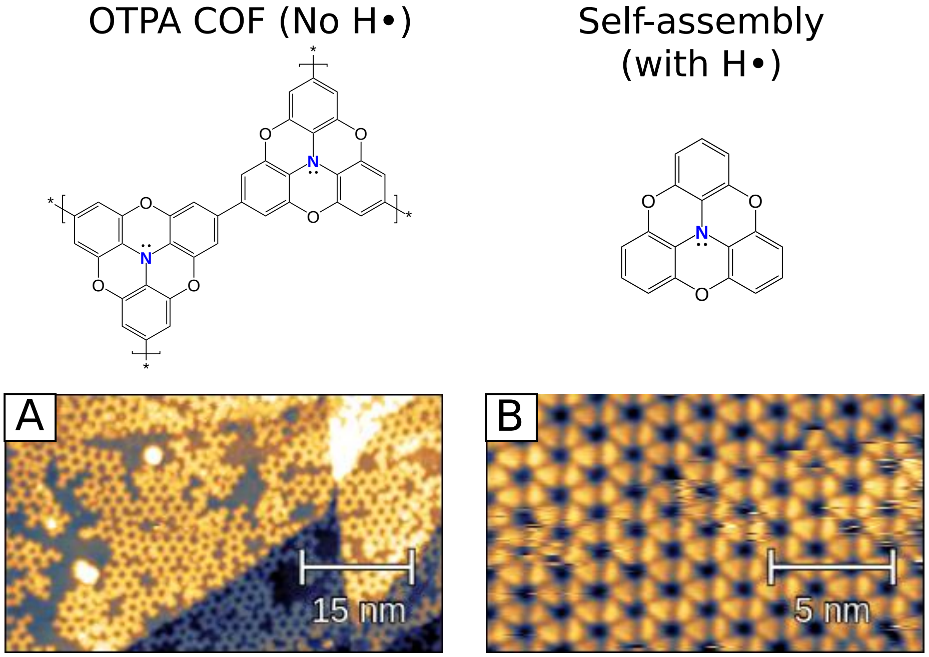

Figure S10: Results of OTPA deposition on Au(111) substrate. (A) 473 K(est.) substrate, in 3×10^{-8} mbar of background H_2 (B) 453 K(est.) in 2.2×10^{-6} mbar of background H_2 and hot ion gauge filament with an emission current 0.1 mA. Imaging parameters: (A) 0.68 V, 130 pA and (B) 1.83 V, 24 pA.

ToF-SIMS: Deuterium substitution

We also perform a deposition of (Br_3)OTPA molecules on a heated Ag(111) substrate in the presence of atomic deuterium (backfilling the chamber with deuterium gas along with a hot ion gauge filament for cracking) and perform ToF-SIMS on the sample, following the same procedure as described in the main paper. We compare the mass spectra of the hydrogenated OTPA on Au(111) sample with the deuterated OTPA on Ag(111) sample (Figure S11). The primary peak in the deuterated sample is shifted up by 3u (due to the heavier deuterium atoms). In addition to the fully deuterated peak corresponding to D_3-OTPA, we also observe mass peaks that consist of partially deuterium and hydrogen substituted species (HD_2-OTPA and H_2D-OTPA) as well as a peak corresponding to the fully hydrogenated OTPA. We attribute these peaks to the existence of background hydrogen in the chamber in addition to the deuterium that has been introduced (although, we don't exclude the possibility of minor H-D substitution during the 5-min atmospheric exposure during transfer to the TOF-SIMS instrument). Using the areas of the peaks, we estimate that $\approx 68.7\%$ of the sites are passivated by deuterium, the rest being passivated by hydrogen. The set of four additional peaks at masses 292.7-298.7 are attributed to the Ag_2Br^+ ions that occur due to bromine adsorbed on the silver surface.

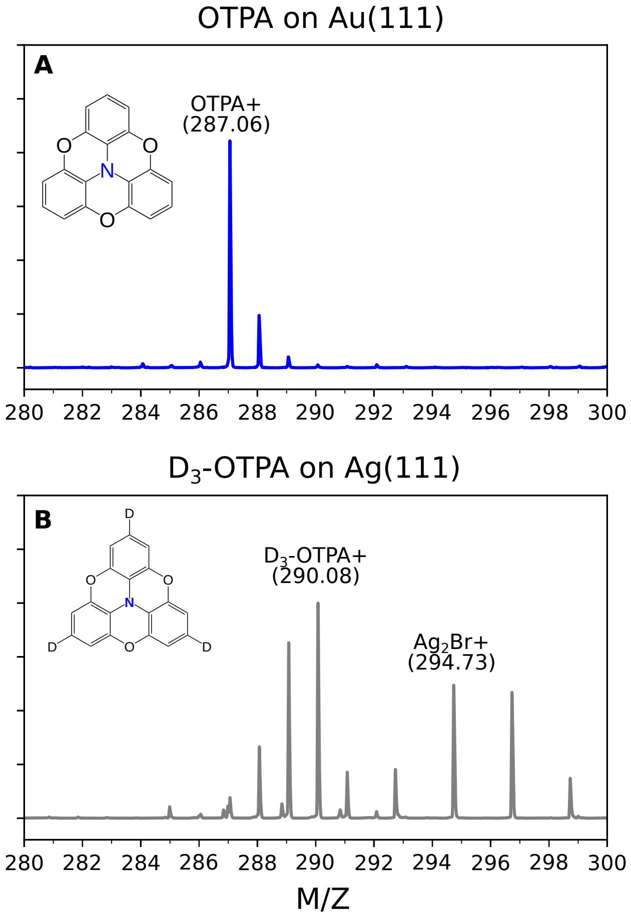

Figure S11: Comparison of the OTPA on Au(111) spectrum (A) and OTPA on Ag(111) spectrum (B). The primary peak in (B) matches the expected mass of the deuterium-terminated OTPA (290.08), providing further evidence of our proposed termination. The cluster of four peaks (292.73, 294.73, 296.73, 298.73) are attributed to Ag_2Br^+ formed by the surface bromine that is released during dehalogenation of (Br_3)OTPA.

Hence, this experiment confirms that the source of the hydrogenation of the deposited molecules is the background gas during deposition, as opposed to other sources or atmospheric contamination.

DTPA Commensurate Overlayer

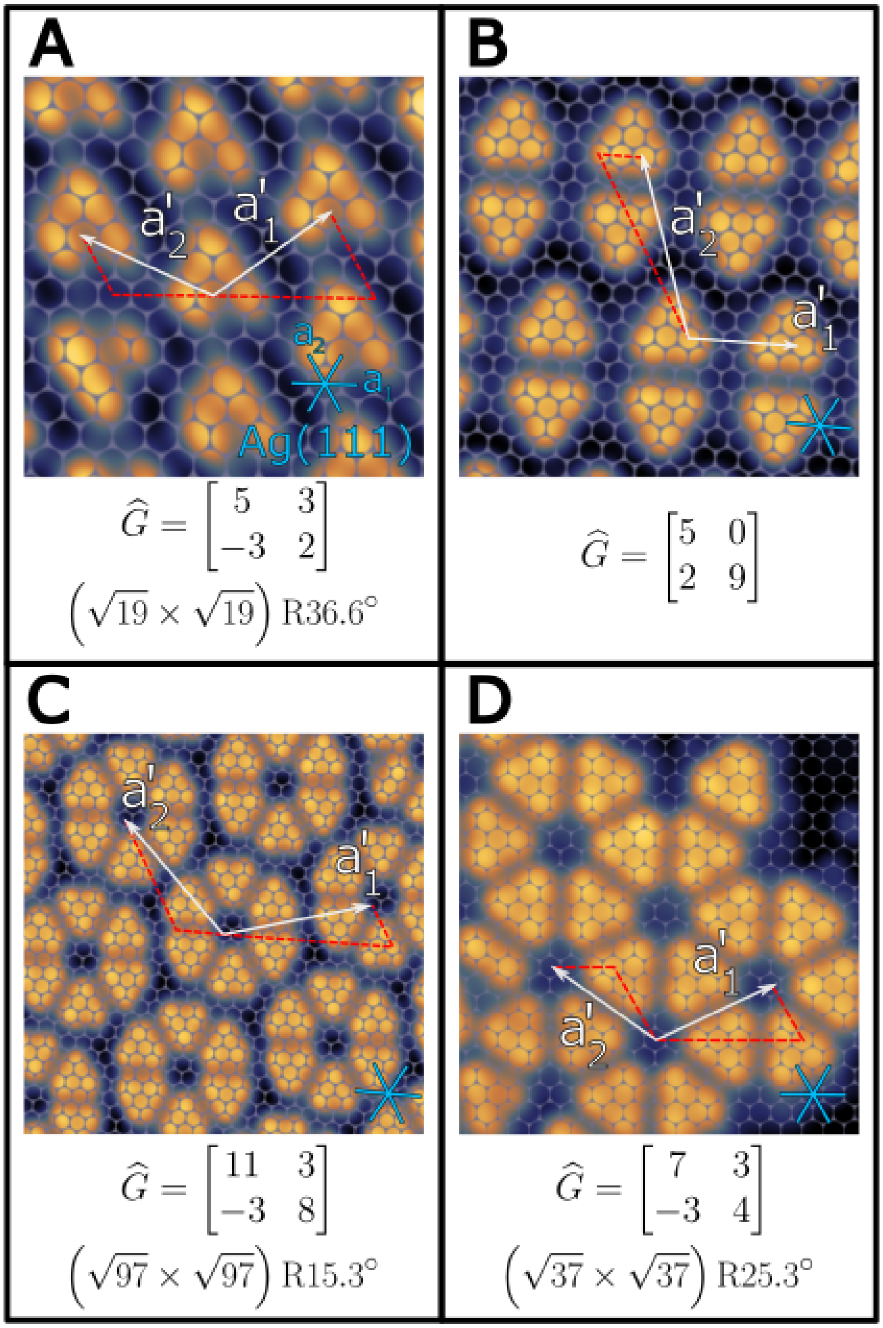

Figure S12: STM topographs of DTPA commensurate molecular overlayers on a synthetic Ag(111) lattice (hexagonal lattice with $a_{Ag(111)} = 0.288\,\text{nm}$). Topographs feature overlayer lattice vectors ($\vec{a}'_1$ and $\vec{a}'_2$) with white arrows and their components projected onto the Ag(111) lattice by the red dashed lines. Below the topographs are the matrix and Wood's notation for the commensurate overlayer structures. (A) DTPA monomer SAM. (B) DTPA dimer SAM. (C) DTPA hexamer macrocycle SAM. (D) DTPA COF.

In Figure S12, a synthetic Ag(111) lattice is superimposed over STM topographs of SAM structures (to avoid obscuring the molecules, the shaded honeycomb denotes the *interstitial* regions of the Ag(111) surface; Ag atom positions are the transparent circles). Although the Ag lattice was generally not resolved while imaging molecular islands, its orientation was determined by Ag $\langle 1\bar{1}0 \rangle$-aligned step-edges in larger scans over the same region (a minimum of two step directions among $[1\bar{1}0]$, $[10\bar{1}]$, or $[01\bar{1}]$ was used). Without atomic resolution of the Ag lattice, the precise location of a molecule over the substrate was not observed experimentally. The lateral alignment of the lattice shown in Figure S12 is chosen to align the DTPA N-centers over 3-fold hollow sites, a configuration which is consistent with the lowest-energy configuration of a closely-related CH_2-bridged TPA COF on the Ag(111) surface, calculated for another purpose (Figure S13).

DFT Adsorption Calculations

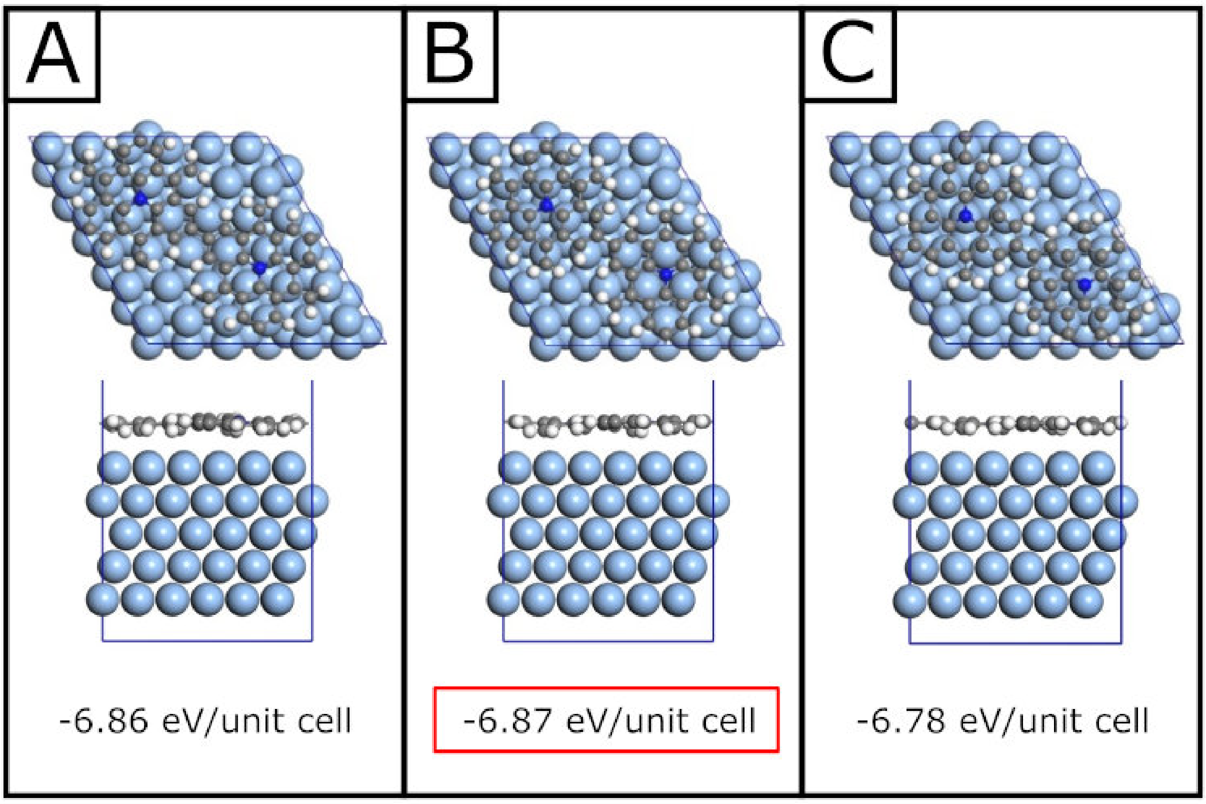

Figure S13: The interface system of CH_2-bridged DTPA-COF on the Ag substrate was calculated at the DFT-PBE level of theory (see Methodology in the main text). Three adsorption sites, denoted as (A) bridge, (B) hollow, and (C) top sites and characterized by the relative positions of the center-N atom in the DTPA COF were considered in the calculations. The relative stability of the three adsorption sites was characterized by comparing their adsorption energies, defined as $E_{ads} = E_{total} - E_{Ag} - E_{COF}$, where E_{total}, E_{Ag}, and E_{COF} are the energies of COF/Ag complex, the Ag slab, and the COF monolayer, respectively.

Tight binding calculations

We consider a simple six-member ring tight binding Hamiltonian with nearest neighbor and next-nearest neighbor terms to model the evolution of the filled state energy levels from monomer to six-membered macrocycle as shown in Figure S14. Since all the sites of the six membered ring are equivalent, we ignore their on-site energies.

$$\mathcal{H} = \sum_{nn} t_{nn}(a_i^\dagger a_j + a_j^\dagger a_i) + \sum_{nnn} t_{nnn}(a_i^\dagger a_j + a_j^\dagger a_i)$$

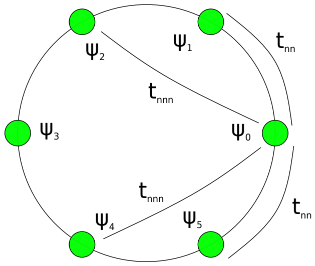

Figure S14: Graphical representation of the tight-binding network used in the case of a 6-membered ring. All (nearest neighbor (nn) and next-nearest neighbor (nnn) are shown for one site (ψ_1).

Now, we consider a ring with N orbital sites.

$$t_{nn}(\psi_{n-1} + \psi_{n+1}) + t_{nnn}(\psi_{n-2} + \psi_{n+2}) = E\psi_n$$

Here, ψ_n refers to the amplitude of the wavefunction at site n and the indices n wrap around the N member circle.

This admits solutions of the form $\psi_n = e^{\frac{i2\pi mn}{N}}$, where $m \in \{0, 1, ..., N-1\}$, with solutions $E_m = 2t_{nn}cos\left(\frac{2\pi m}{N}\right) + 2t_{nnn}cos\left(\frac{4\pi m}{N}\right)$. For the six-membered ring system which we use, the solutions are singly degenerate levels at $(2t_{nn} + 2t_{nnn}, -2t_{nn} + 2t_{nnn})$ and doubly degenerate levels at $(t_{nn} - t_{nnn}, -t_{nn} - t_{nnn})$.

Comparing the peak locations predicted by this model to the DFT peak locations, we obtain $t_{nn} \simeq -0.2\,\text{eV}$ and $t_{nnn} \simeq -0.012\,\text{eV}$.

Freestanding DTPA COF DFT Bandstructure

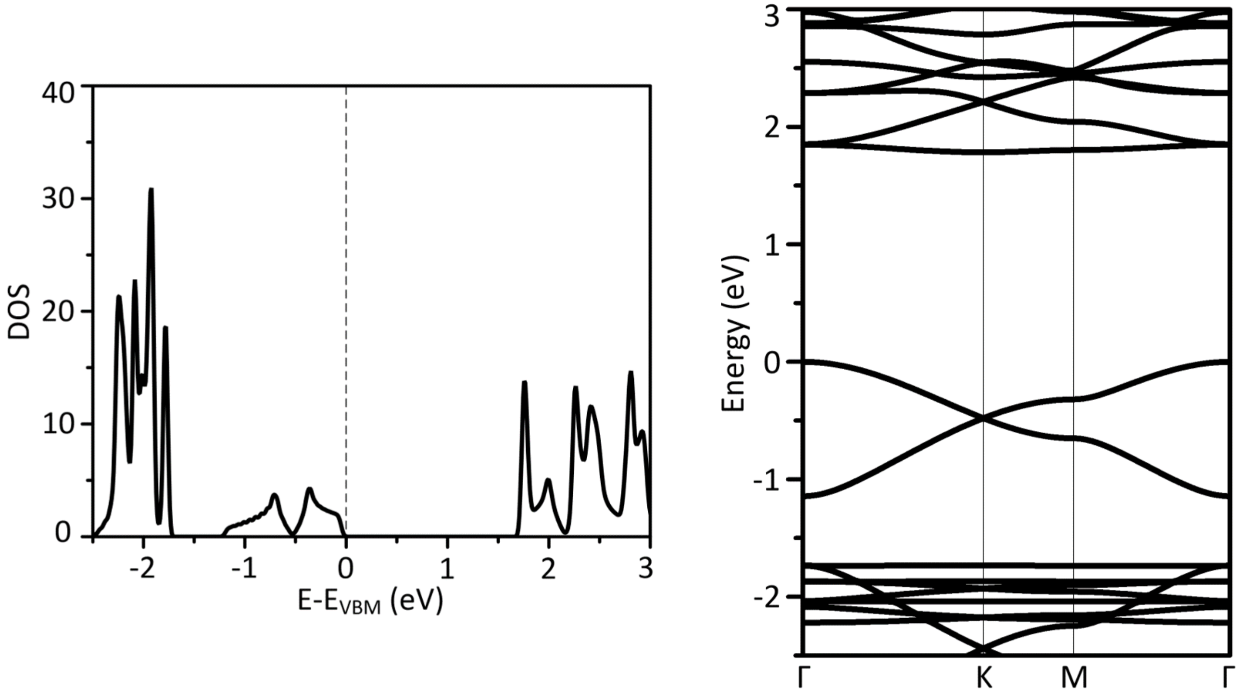

Figure S15: Left: DFT calculated density of states for the freestanding DTPA COF. Right: DFT calculated bandstructure for the freestanding DTPA COF.

Spectral Map Analysis

Figure S16 shows the data analysis steps underlying Figure 6 of the main text.

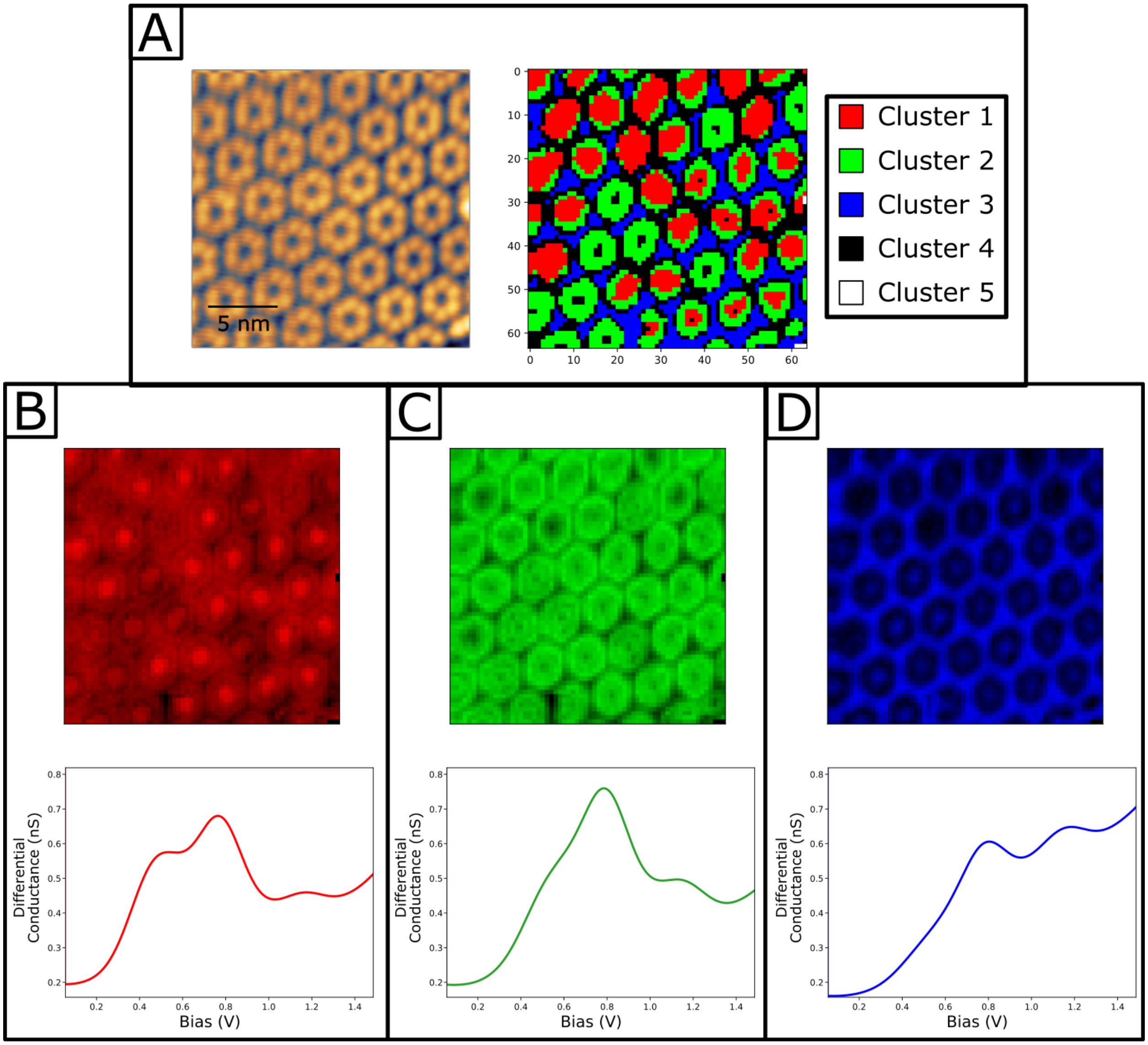

Figure S16: Details on the spectral map analysis using the hexamer SAM data as an example. (A) A reference topograph taken over the hexamer SAM immediately prior to the spectral map acquisition and the sorted results from K-means clustering using five clusters. The K-means results display the 64x64 pixel map where the scanning tunneling spectra are taken and each color designates its cluster. (B-D) Representation of the vector 2-norm in the vector space spanned by the bias voltages with the displayed spectra as the origin. These spectra are the average differential conductance spectra between 0.05 V to 1.5 V from the selected clusters. The Euclidean distances were used to generate the red (B), green (C), and blue (D) pixel values in middle column of Figure 6.

The spectral map data is first sorted by K-means clustering into 5 clusters. K-means was chosen because it can sort the spectral data without the necessity for user intervention or thresholding. K-means is performed on the differential conductance spectra in the range from 0.05 V to 1.5 V which has been normalized by its integral value. Representative spectra are chosen as the origin to calculate a Euclidean distance in the vector space spanned by the bias voltages. These representative spectra are the averaged spectra from cluster 1 (B), cluster 2 (C) and cluster 3 (D). The clusters were chosen based on the spatial portion of the molecular formation under investigation. The Euclidean distance is the vector 2-norm from the spectrum at a pixel location in the spectral map to the cluster averaged spectrum (cluster center) resulting in three Euclidean distances per pixel location. These values are normalized by the range of values (per spectral origin point) and subtracted by 1 resulting in a value between 0-1 with 1 being closest to the referenced spectrum and 0 furthest away. The sets of these values are shown in the maps presented in (B-D). These maps are converted into the RGB values displayed in Figure 6 with the red pixel value from map (B), green from map (C), and blue from map (D).

Freestanding DTPA DFT Partial Charge Density Maps

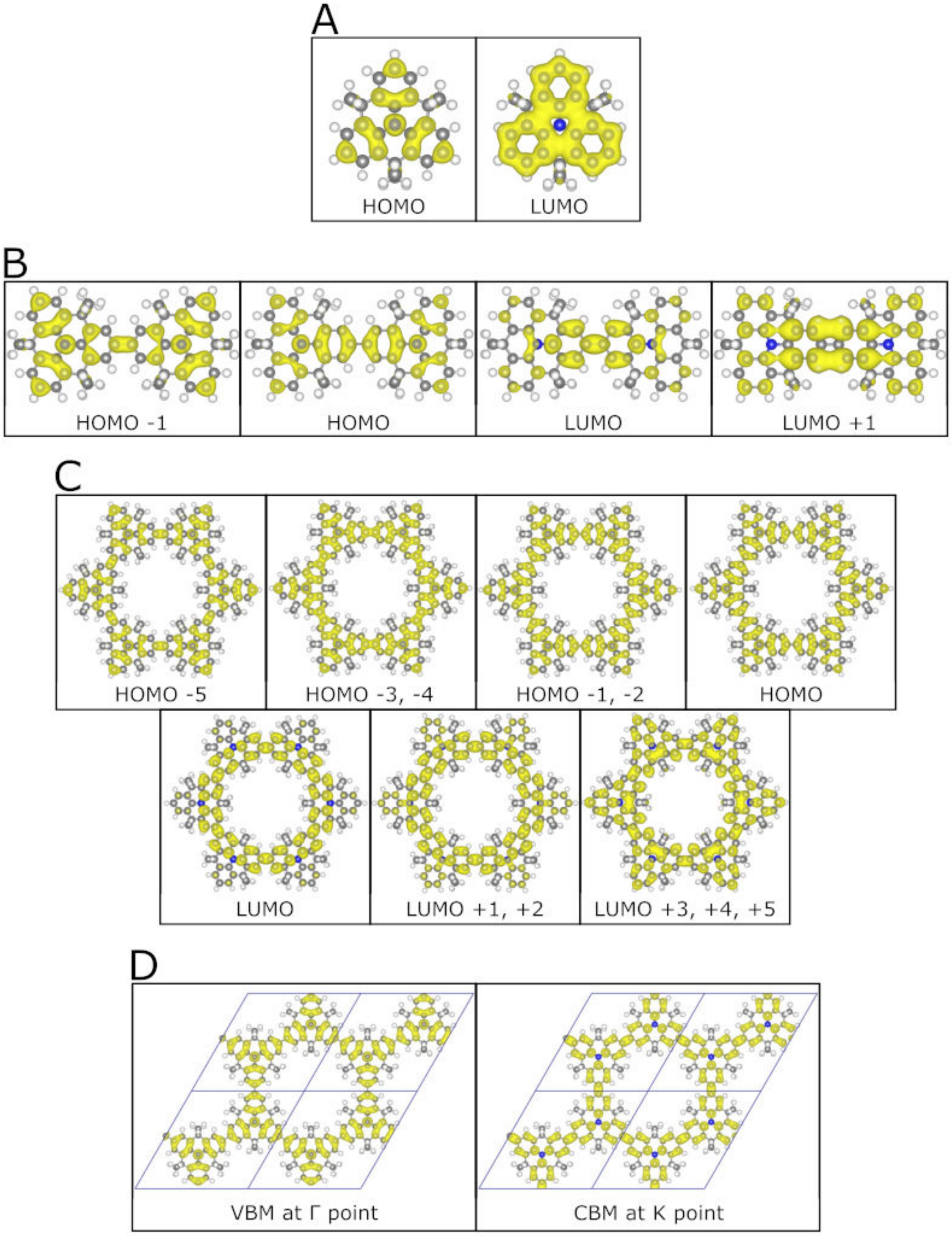

Figure S17: DTPA charge density maps calculated by freestanding DFT for various DTPA structures and molecular orbitals/bands. (A) DTPA monomer. (B) DTPA Dimer. (C) DTPA hexamer macrocycle. (D) DTPA COF.

Electron confined to a circle in two dimensions

Consider an electron obeying the Schrödinger equation confined to a circle in two dimensions with radius ρ.[2]

$$\frac{-\hbar^2}{2m^*}\nabla^2\psi = E\psi$$

Expanding ∇^2 in polar coordinates, we get:

$$\nabla^2 = \frac{\partial^2}{\partial r^2} + \frac{1}{r}\frac{\partial}{\partial r} + \frac{1}{r^2}\frac{\partial^2}{\partial \theta^2}$$

Splitting ψ as $R(r)\Phi(\theta)$,

$$\frac{r^2}{R}\frac{\partial^2 R}{\partial r^2} + \frac{r}{R}\frac{\partial R}{\partial r} + \frac{2m^*E}{\hbar^2}r^2 = m^2 \tag{1}$$

where $m \in \mathbb{Z}$ due to the condition on the angular part of the wavefunction ($\Phi(\theta+2m\pi) = \Phi(\theta)$).

Substituting $r' = \sqrt{\frac{2mE}{\hbar^2}}r$, we get the Bessel differential equation:

$$r'^2\frac{\partial^2 R}{\partial r'^2} + r'\frac{\partial R}{\partial r'} + R(r'^2 - m^2) = 0 \tag{2}$$

The solutions of this equation are the Bessel functions. If we constrain the solutions of this equation to be zero at $r = \rho$ and that $R(0)$ isn't a singularity, $R(r')$ at $r = \rho$ must be zero. The lowest energy mode would correspond to the location of the first zero of $J_0(r')$,[2] ie.,

$$E = E_0 + \frac{\hbar^2}{2m^*}\left(\frac{2.4048}{\rho}\right)^2$$

Since we are looking at the confinement of surface state electrons with parabolic dispersion, we add the energy term E_0, where E_0 is the onset of the surface state.

If we take $m^* = 0.42m_e$ and an approximate radius of the confinement provided by the hexamers/COF as $\rho = 0.94\,\text{nm}$ and $E_0 = -50\,\text{meV}$, we get a ground state energy of

$E = 0.54\,\text{eV}$, which approximates the pore resonance observed in the EPWE model.

Electron Plane Wave Expansion modeling

The free surface state electrons obey a parabolic energy dispersion given by:

$$E_k = E_0 + \frac{\hbar^2 k^2}{2m^*}$$

Here, E_0 is the surface state band onset energy (measured to be $-50\,\text{meV}$ from STS) and m^* is the effective mass of the surface state electrons, $m^* = 0.42m_e$.[3]

The adsorbed molecules on the metal surface can be modelled as regions containing a scattering potential that affects the two dimensional surface electron gas.[4–6] The potentials can be used to set up an effective Schrödinger equation. The differential equation can be solved numerically using a variety of methods, such as the Boundary Element Method[4] or an Electron Plane Wave Expansion (EPWE) method.[7,8]

In the EPWE method,[7] we consider the Schrödinger equation with a periodic potential V:

$$\left(\frac{-\hbar^2\nabla^2}{2m^\star} + V(\mathbf{r})\right)\psi = E\psi$$

The potential V can be expanded in two dimensions using a Fourier series summation as $V(\mathbf{r}) = \sum_{\mathbf{G}} V_{\mathbf{G}} e^{i\mathbf{G}.\mathbf{r}}$, where the summation is over all the reciprocal lattice vectors $\mathbf{G}$ of the periodic potential V. We can also expand the Bloch wavefunction $\psi = u(\mathbf{r})e^{i\mathbf{k}.\mathbf{r}}$ where $u(\mathbf{r})$ has the same periodicity as the potential $V(\mathbf{r})$ and $\mathbf{k}$ is a vector in the first Brillouin zone. Hence, the wavefunctions ψ can also be expanded in terms of a Fourier series of the reciprocal lattice as $\psi(\mathbf{r}) = \sum_{\mathbf{G'}} u_{\mathbf{G'}} e^{i(\mathbf{G'}+\mathbf{k}).\mathbf{r}}$. We insert these terms into the equation. Then, for every reciprocal lattice vector $\mathbf{G}_0$, we can write:

$$\frac{\hbar^2}{2m^*}(\mathbf{G}_0 + \mathbf{k})^2 u_{\mathbf{G}_0} + \sum_{\mathbf{G}} V_{\mathbf{G}_0-\mathbf{G}} u_{\mathbf{G}} = E u_{\mathbf{G}_0}$$

While this equation is for all reciprocal lattice vectors $\mathbf{G}_0$, for the purpose of the numerical calculation, we restrict the set of lattice vectors to within a circle in the reciprocal space with a radius sufficiently large (with sufficiently many Fourier components) to describe the potential accurately and obtain convergence. Hence, this equation can be written as an eigenvalue equation $\mathcal{H}U = EU$, where the matrix $\mathcal{H}_{ij} = V_{\mathbf{G}_i - \mathbf{G}_j} + \frac{\hbar^2}{2m^*}(\mathbf{G}_i + \mathbf{k})^2\delta_{ij}$. This eigenvalue equation can be solved numerically for $\mathbf{k}$ to obtain the wavefunction energies and densities.

To approximate the DTPA molecules, we constructed a model potential of Gaussian potentials, $V(\mathbf{r}) = We^{\frac{-\mathbf{r}^2}{2\sigma^2}}$, at every atomic site of the DTPA π-skeleton. For these computations, we choose $W = 0.5\,\mathrm{eV}$ and $\sigma = 0.7\,\text{Å}$.

Using these model molecular potentials, we can calculate the LDOS of the surface state electrons with confining hexamer as well as COF potentials, as shown in Figure S18. For this calculation, we consider all the reciprocal lattice vectors $\mathbf{G}$ that are inside a circle of radius $15|\mathbf{k}_0|$, where $|\mathbf{k}_0|$ is the magnitude of the reciprocal lattice vectors of the lattices being considered (COF and hexamer) in order to accurately capture the potential. We truncate the higher order Fourier components since they do not contribute significantly to the low energy confinement LDOS. The LDOS is then calculated over one rhombus shaped lattice tile enclosed by the lattice vectors $\mathbf{a}_1$ and $\mathbf{a}_2$ in a 50×50 grid. The rhombus tile is then repeated to tile the image shown in Figure S18, and the spectra at points of interest are extracted by linear interpolation within the tiled set of spectra. The calculated LDOS spectra are then smoothed in energy using a gaussian filter with a standard deviation of $8.5\,\mathrm{meV}$ to reflect the experimental broadening.

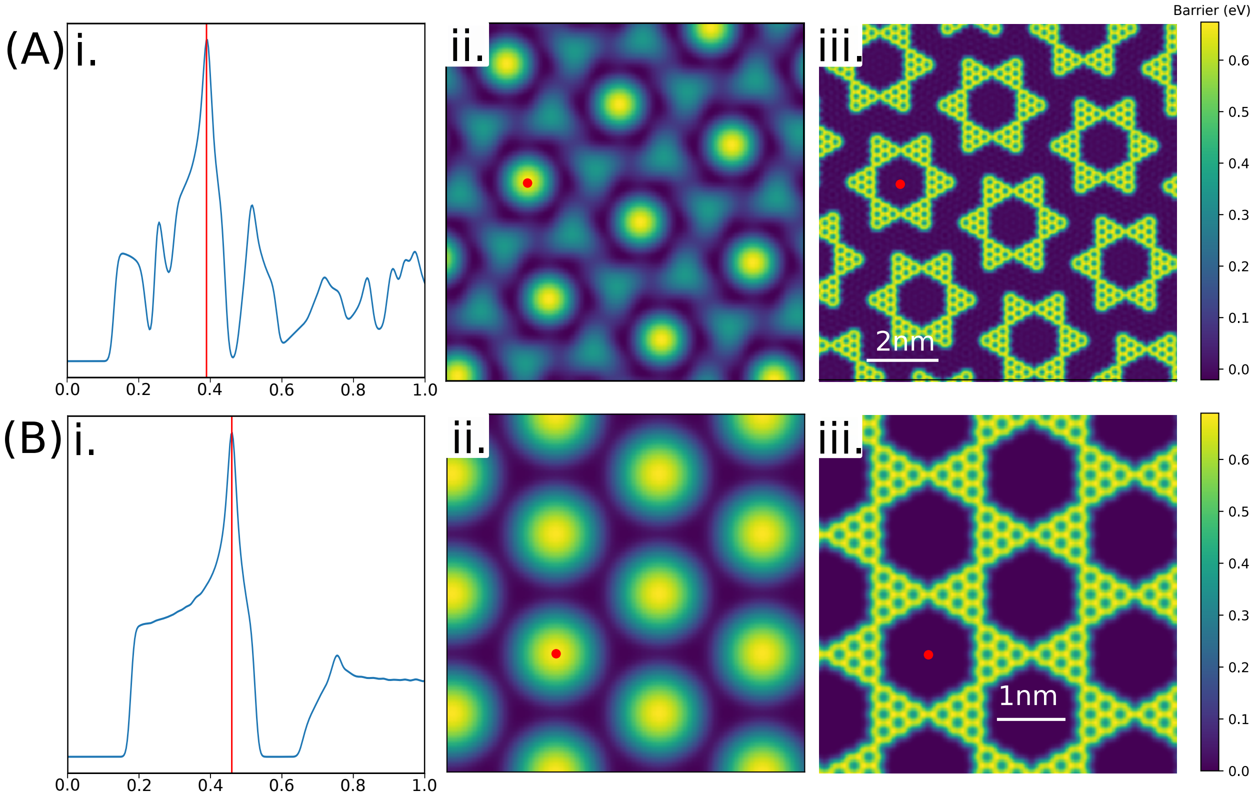

Figure S18: The calculated (i) LDOS at pores, (ii) LDOS intensity map for the pore resonance peak and (iii) Potential used for EPWE calculations for (A) Hexamer self-assembly and (B) COF respectively. The red line in (i) marks the energy of the calculated LDOS map. The red point in (ii),(iii) shows the location (inside the pores) of the spectrum shown in (i). The spectra are broadened in energy with a gaussian kernel ($\sigma = 8.5\,\text{meV}$).

The pore spectrum of the hexamer shows a peak with a maximum at 0.39 eV, while the pore spectrum of the COF shows a maximum at 0.46 eV. The LDOS maps plotted at these voltages show that the intensity is concentrated at the pores, indicating that this peak corresponds to a pore resonance. The LUMO states also have higher density towards the pores (Figure S17), which contributes to the higher density of states towards the pore regions. This matches with the experimental observation of increased differential conductance at the pore centers in the case of both hexamer and COF spectra in the region of $\sim 0.5\,\text{eV}$.

References

1. Marchi, F. D.; Galeotti, G.; Simenas, M.; Gallagher, M. C.; Hamzehpoor, E.; MacLean, O.; Rao, R. M.; Chen, Y.; Dettmann, D.; Contini, G.; Tornau, E. E.; Ebrahimi, M.; Perepichka, D. F.; Rosei, F. Temperature-Induced Molecular Reorganization on Au(111) Driven by Oligomeric Defects. *Nanoscale* **2019**, *11*, 19468–19476, DOI: 10.1039/C9NR06117G.

2. Crommie, M. F.; Lutz, C. P.; Eigler, D. M.; Heller, E. J. Quantum Corrals. *Phys. D: Nonlinear Phenom.* **1995**, *83*, 98–108, DOI: 10.1016/0167-2789(94)00254-N.

3. Li, J.; Schneider, W.-D.; Berndt, R.; Crampin, S. Electron Confinement to Nanoscale Ag Islands on Ag(111): A Quantitative Study. *Phys. Rev. Lett.* **1998**, *80*, 3332–3335, DOI: 10.1103/PhysRevLett.80.3332.

4. Kepčija, N.; Huang, T.-J.; Klappenberger, F.; Barth, J. V. Quantum Confinement in Self-assembled Two-Dimensional Nanoporous Honeycomb Networks at Close-Packed Metal Surfaces. *J. Chem. Phys.* **2015**, *142*, 101931, DOI: 10.1063/1.4913244.

5. Klappenberger, F.; Kühne, D.; Krenner, W.; Silanes, I.; Arnau, A.; García de Abajo, F. J.; Klyatskaya, S.; Ruben, M.; Barth, J. V. Dichotomous Array of Chiral Quantum Corrals by a Self-Assembled Nanoporous Kagomé Network. *Nano Lett.* **2009**, *9*, 3509–3514, DOI: 10.1021/nl901700b.

6. Klappenberger, F.; Kühne, D.; Krenner, W.; Silanes, I.; Arnau, A.; García de Abajo, F. J.; Klyatskaya, S.; Ruben, M.; Barth, J. V. Tunable Quantum Dot Arrays Formed from Self-Assembled Metal-Organic Networks. *Phys. Rev. Lett.* **2011**, *106*, 026802, DOI: 10.1103/PhysRevLett.106.026802.

7. Abd El-Fattah, Z. M.; Kher-Elden, M. A.; Piquero-Zulaica, I.; de Abajo, F. J. G.; Ortega, J. E. Graphene: Free Electron Scattering within an Inverted Honeycomb Lattice. *Phys. Rev. B* **2019**, *99*, 115443, DOI: 10.1103/PhysRevB.99.115443.

8. Kawai, S.; Kher-Elden, M. A.; Sadeghi, A.; Abd El-Fattah, Z. M.; Sun, K.; Izumi, S.; Minakata, S.; Takeda, Y.; Lobo-Checa, J. Near Fermi Superatom State Stabilized by Surface State Resonances in a Multiporous Molecular Network. *Nano Lett.* **2021**, *21*, 6456–6462, DOI: 10.1021/acs.nanolett.1c01200.
